# Comprehensive analysis of the REST transcription factor regulatory networks in IDH mutant and IDH wild-type glioma cell lines and tumors

**DOI:** 10.1186/s40478-024-01779-y

**Published:** 2024-05-06

**Authors:** Malgorzata Perycz, Michal J. Dabrowski, Marta Jardanowska-Kotuniak, Adria-Jaume Roura, Bartlomiej Gielniewski, Karolina Stepniak, Michał Dramiński, Iwona A. Ciechomska, Bozena Kaminska, Bartosz Wojtas

**Affiliations:** 1 Laboratory of Molecular Neurobiology, Nencki Institute of Experimental Biology, Polish Academy of Sciences, Warsaw, Poland; 2 Computational Biology Group, Institute of Computer Science of the Polish Academy of Sciences, Warsaw, Poland; 3 Doctoral School of Institute of Biochemistry and Biophysics, Polish Academy of Sciences, Warsaw, Poland; 4 Laboratory of Sequencing, Nencki Institute of Experimental Biology, Polish Academy of Sciences, Warsaw, Poland

**Keywords:** Differentiation, Glioblastoma, *IDH* mutation, REST, KAISO, ZBTB33, DNA methylation, Transcription factor, Extracellular matrix, Invasion

## Abstract

The RE1-silencing transcription factor (REST) acts either as a repressor or activator of transcription depending on the genomic and cellular context. REST is a key player in brain cell differentiation by inducing chromatin modifications, including DNA methylation, in a proximity of its binding sites. Its dysfunction may contribute to oncogenesis. Mutations in *IDH1/2* significantly change the epigenome contributing to blockade of cell differentiation and glioma development. We aimed at defining how REST modulates gene activation and repression in the context of the *IDH* mutation-related phenotype in gliomas. We studied the effects of REST knockdown, genome wide occurrence of REST binding sites, and DNA methylation of REST motifs in *IDH* wild type and *IDH* mutant gliomas. We found that REST target genes, REST binding patterns, and TF motif occurrence proximal to REST binding sites differed in *IDH* wild-type and mutant gliomas. Among differentially expressed REST targets were genes involved in glial cell differentiation and extracellular matrix organization, some of which were differentially methylated at promoters or gene bodies. REST knockdown differently impacted invasion of the parental or *IDH1* mutant glioma cells. The canonical REST-repressed gene targets showed significant correlation with the GBM NPC-like cellular state. Interestingly, results of REST or KAISO silencing suggested the interplay between these TFs in regulation of REST-activated and repressed targets. The identified gene regulatory networks and putative REST cooperativity with other TFs, such as KAISO, show distinct REST target regulatory networks in IDH-WT and IDH-MUT gliomas, without concomitant DNA methylation changes. We conclude that REST could be an important therapeutic target in gliomas.

**Supplementary Information:**

The online version contains supplementary material available at 10.1186/s40478-024-01779-y.

## Introduction

REST (RE1-silencing transcription factor), previously known as NRSF – the neuron-restrictive silencer factor, together with its co-repressor complex participates in shaping neuronal differentiation in the developing brain and represses neuronal gene expression in non-neuronal terminally differentiated cells [1]. REST activity has been reported to contribute to numerous diseases, although its role is divergent [2]. REST acts as a tumor suppressor in epithelial cancers [3–5] and as an oncogene in childhood brain neoplasms such as neuroblastoma and medulloblastoma [6, 7]. DNA methylation within the predicted REST binding sites was shown to have a prognostic value for glioma patients overall survival [8]. Moreover, increased REST activity, described with gene expression-based signature of 77 REST targets, correlated with worse clinical outcome [9].

Mutations in genes coding for IDH1 or IDH2 (isocitrate dehydrogenases) occur frequently in oligodendroglioma WHO grades 2, 3 and astrocytoma grades 2, 3, 4 resulting in global changes in DNA methylation. A mutated IDH enzyme, most frequently carrying a R132H substitution, catalyzes the production of not only its canonical product α-ketoglutarate (α-KG) but also 2-hydroxyglutarate oncometabolite (2-HG), which inhibits many epigenetic, α-KG dependent enzymes, including DNA and histone demethylases [10, 11]. As DNA and histone demethylation are inhibited, cells are blocked in the hypermethylated epigenetic state, which dysregulates expression of genes and blocks cell differentiation [12, 13]. Whole genome epigenetic changes related to *IDH1/2* mutations are often described as the IDH-related phenotype [14].

DNA methylation changes can modulate the binding of transcription factors [15]. DNA methylation changes in both CpG [1, 16] and non-CpG context [17] impacted REST binding affinity and its target genes’ expression [17]. REST binds to several DNA motifs that can be either methylated or not [18]. Moreover, REST can modulate DNA methylation in the close proximity of its binding [19]. Upon binding to DNA, REST can recruit many epigenetic factors that lead to imprinting of active or repressive epigenetic marks. In most cases when REST binds to its canonical RE1 sites, it results in repression of its targets [1, 20, 21]. REST can bind to non-canonical sites [1] but the effect on gene activation or repression is not well described.

Removal of REST in neural progenitors reduced their proliferation [22]. Intriguingly, the expression of many classic REST target genes were not significantly changed, while groups of genes that are not normally regulated by REST were activated, including the p53 pathway. Additional removal of p53 in REST-null mice led to development of the proneural type glioblastoma (GBM) with primitive neuroepithelial tumors (PNET) characteristics in mice [22]. In GBM stem cells REST maintains oncogenic properties and self-renewal [23, 24]. Increased REST expression decreased apoptosis and increased invasive properties of GBM cells [23], while knockdown of REST in GBM xenografts led to either cell differentiation or cell death significantly impairing tumor growth [24]. siRNA mediated knockdown of REST in established human U-87 MG and U-251 glioma cells reduced cell proliferation and migration [25].

In the present study, we assessed whether repressing and activating functions of REST are modified by the IDH-related phenotype in gliomas and U-87 MG glioma cell lines, the only established glioma cell line present as an isogenic pair differing just in the IDH1 status. We found that genome-wide REST binding patterns and TF motif occurrence at the REST binding sites were different in IDH wild-type and IDH mutant gliomas. Genes involved in glial cell differentiation and extracellular matrix (ECM) organization were among differentially methylated REST targets. REST knockdown differently affected cell invasion of the parental or *IDH1-*mutated glioma cells. Genes identified as REST-repressed in siREST experiment correlated with the NPC-like cellular state in GBM scRNA-seq data [26]. The occurrence of REST ChiP-seq peaks specifically in IDH-WT or IDH-MUT was not associated with DNA methylation of the loci. Regulatory sequences of REST-repressed and REST-activated genes showed different TF motif composition and DNA methylation patterns. We found overlaps of REST and KAISO (coded by *ZBTB33* gene) regulatory networks suggesting synergistic roles in transcriptional repression and competing roles in transcriptional activation. The identified REST targets, gene regulatory networks and putative REST cooperativity with other TFs, including KAISO, point to distinctiveness of the REST regulome in IDH-WT and IDH-MUT gliomas. Our findings contribute to better predictions of glioma patient survival based on DNA methylome and transcriptome evaluation, and suggest a potential of targeting REST in glioma therapy.

## Materials and methods

### Cell culture

The experiments were conducted using U-87 MG (ATCC HTB-14 IDH-WT) and *IDH1* mutant-U-87 Isogenic Cell Line (ATCC HTB-14IG), hereinafter referred to as U87 IDH-WT and U87 IDH-MUT, purchased from American Type Culture Collection (ATCC). The *IDH1* mutant isogenic U87 cells (ATCC cat.no HTB-14IG) carry a heterozygous   C395G > A mutation (IDH1R132H). The *IDH1* mutant-U87 isogenic cell line has been widely used in glioma research (https://www.atcc.org/products/htb-14ig). The cells were cultured in DMEM (Gibco), 10% FBS (PAN-BIOTECH) with no antibiotics added. Cells were passaged every 3–5 days depending on the seeding density.

### Human glioma samples

This study used glioma datasets generated by Stepniak et al., 2021 [27] (EGA accession nr: ERP125425), hereinafter referred to as “glioma Atlas”.

### REST-ChIP-seq in human glioma samples

The results of REST chromatin immunoprecipitation followed by sequencing (ChIP-seq) on freshly resected gliomas (n = 7) were obtained with a described protocol [27] with the exception of using the REST antibody (Millipore, CS200555) instead of a histone mark antibody.

### Human glioma samples: methylomes

5mC DNA methylation data (NimbleGen SeqCap Epi from ROCHE) include glioma tumor samples of WHO grade 2/3 IDH-WT (n = 4), grade 4 IDH-WT (n = 11) and four IDH-MUT samples (n = 3 G2/3; n = 1 G4 astrocytoma), obtained from the recently published glioma Atlas [27]. All four IDH-MUT samples were pooled together and hereafter are referred to as IDH-MUT. The methylomes of glioma Atlas cover millions of cytosines at a base pair resolution. Due to tumor-related DNA degradation, methylomes presented in glioma Atlas consist of over 10 million of cytosines per sample on average. Despite the limited number of tumor type specific samples, the number of covered DNA sites in glioma Atlas is a huge advantage in comparison to BeadChip panels such as Infinium® HumanMethylation450 or MethylationEPIC performed on larger patient cohorts.

### Human glioma samples: RNA-seq

RNA-seq row counts of genes were obtained for the same set of samples from the glioma Atlas whose methylomes were analyzed (n = 4 IDH-MUT, n = 4 IDH-WT LGG (lower grade gliomas, WHO grade 2/3), n = 11 IDH-WT G4) [27]. Two normalization procedures were applied: (i) within-lane to adjust for GC-content and gene-length; (ii) between-lane, both implemented in EDASeq 3.8 R package [28]. The normalized gene expressions were used to investigate correlation of DNA methylation level with gene expression.

### TCGA public data

TCGA level 3 RNA-seq data repositories of GBM (glioblastoma, WHO grade 4) and LGG samples, aligned by STAR and gene expression counted by HTseq, (https://portal.gdc.cancer.gov/) were uploaded to R. Gene expression levels as FPKM (fragments per kilobase of exon per million) were used for further analysis of REST expression in the context of glioma malignancy grades (G2 n = 248, G3 n = 261, G4 n = 160) and *IDH1/2* mutation status (G2/G3 IDH-MUT n = 365, G2/G3 IDH-WT n = 79). Clinical data for LGG and GBM datasets were obtained from [29] (Additional file 11: Table S1).

### Grade 4 primary glioma cell lines

RNA-seq results (DESeq2 normalized) from 12 grade 4 glioma cell lines, 2 IDH-MUT astrocytomas and 10 GBM IDH-WT [30] were used for U87 IDH-MUT model validation in primary human glioma cell lines.

### Single cell RNA-seq data

Glioma tumor samples scRNA-seq data [26] were uploaded from GEO (GSE131928) and uploaded to R as TPM values.

### siRNA-REST knockdown

REST knockdown was performed in U87 IDH-WT and IDH-MUT, the isogenic malignant glioma cell lines that genetically differed only by *IDH1* mutation status. The cells were subcultured 2 days prior to the transfection so they would not exceed the confluency of 80% on the day of siRNA transfection and double transfected within 24 h, first by nucleofection, followed by lipofection. For the nucleofection, cells were trypsinized, counted, centrifuged, resuspended in Lonza SE cell line solution reagent (Lonza, PBC1-02250) and transferred to Nucleocuvette Vessel (Lonza). Control or human REST-targeting siRNA ON-TARGETplus SMARTpool (Dharmacon, D-001810–10-05 and L-006466–00-0005) was then added to the appropriate wells of the vessel and nucleofection was carried out using 4D-Nucleofector core unit. The cells were then resuspended with DMEM 10% FBS and cultured in 12 or 24 well plates (Falcon) for the next 24 h. After that time, the medium was replaced with fresh DMEM 10% FBS and the cells were transfected for the second time using Lipofectamine 2000 (Invitrogen, 2,094,065) and the same siRNA ON-TARGETplus SMARTpool (Dharmacon) as before. Protein and RNA were collected 72 h after the first transfection.

### siRNA-ZBTB33 (KAISO) knockdown

On-TARGETplus SMARTpool siRNA against human ZBTB33 (siZBTB33, Dharmacon, L-019982–00-0005) or non-targeting control siRNA (siCtrl, Dharmacon, D-001810–10-05) were used. U87 IDH-WT and IDH-MUT glioma cells were cultured in 12-well plates at a density of 8 × 10^4^ cells per well, one day prior to the transfection. Cells were double transfected with 40 nM siRNA within 24 h using Lipofectamine 2000 reagent (Invitrogen, 2,094,065). RNA and protein were collected 72 h after the first transfection. The efficiency of gene knockdown was determined by qPCR and Western blot analysis.

### RNA isolation from U87 cells

RNA was isolated from U87 IDH-WT and IDH-MUT cells that were either not treated or transfected with control or REST-targeting or ZBTB33-targeting siRNA. RNeasy Mini Kit (QIAGEN, 74,106) was used according to the producer’s protocol. RNA concentration was measured with NanoDrop 2000 (Thermo Scientific, NanoDrop products, Wilmington, USA). RNA quality was verified with Bioanalyzer 2100 (Agilent Technologies, Santa Clara, CA) using an RNA 6000 Nano Kit (Agilent Technologies, Ltd., 5067–1511).

### Quantitative PCR (qRT-PCR)

Complementary DNA was synthesized from total RNA by extension of oligo(dT) primers with SuperScript III Reverse Transcriptase (Invitrogen, 18080–044). Real-time PCR was performed applying SYBR Green chemistry (Applied Biosystem by Thermo Fisher Scientific, 4385612) and a set of primers for *REST* (Forward: 5’-GCTGGCAAATGTGGCCTTAAC-3’; Reverse: 5’-AAGTTGTTATCCCCAACCGGC = 3’) on QuantStudio 12 K Flex Real-Time PCR System. Amplified product was normalized to the endogenous expression of *GAPDH* (Forward: 5’-ATCACCATCTTCCAGGAGCGA-3’; Reverse: 5 ‘-AGCCTTCTCCATGGTGGTGAA-3’) and represented as -ΔΔCt (negative delta delta Ct) values (fold change).

Human *ZBTB33* and *MMP7* expression was measured using TaqMan PCR mix (Life Technologies), specific primers and FAM-labelled probe sets (*ZBTB33*—Hs00272725_s1; *MMP7* – Hs01042796_m1) and normalized to *GAPDH* expression (Hs02758991_g1). Data were analysed with the relative quantification (ΔΔCt) method using 7500 System SDS Software (Life Technologies).

Statistical significance of comparisons between groups was calculated in GraphPad Prism v. 9.1.2 (GraphPad Software, LCC). Results were considered significant when *P < 0.05 (Mann–Whitney test). Data were obtained in 4 independent experiments.

### Western Blotting

Cells were lysed, protein concentration was determined, protein extracts were separated by electrophoresis, and transferred to a nitrocellulose membrane (GE Healthcare, 10600003) as described [31]. After blocking with 5% non-fat milk in TBST (Tris-buffered solution pH 7.6, 0.01% Tween-20) the membranes were incubated overnight with primary antibody (rabbit anti-REST1, Millipore CS200555, 1:1000, mouse anti-Kaiso [6F / 6F8], Abcam ab12723, 1:500; rabbit anti-Histone H3 Abcam ab1791, 1:2000; mouse anti-IDH1 R132H, Dianova DIA-H09, 1:500; or mouse anti-GAPDH, Millipore MAB374, 1:500) in TBST with 5% bovine serum albumin (BSA, Sigma) or 1 h with horseradish peroxidase-conjugated anti-β-actin antibody (Sigma, A3854) diluted 1:20,000 in 5% non-fat milk in TBST. The primary antibody reaction was followed by 1 h incubation with horseradish peroxidase- conjugated anti-rabbit IgG diluted at 1:10,000 (Vector, PI-1000). Immunocomplexes were detected with an enhanced chemiluminescence detection system (ECL, BioRad) and Chemidoc (BioRad). The molecular weight of proteins was estimated with Cozy prestained protein ladder (High Qu GmbH, PRL0102c1). Densitometry of band intensities was performed using BioRad Image Lab software. REST band intensities were normalized to GAPDH band intensities for each blot. Statistical significance of comparisons between groups was calculated in GraphPad Prism v. 9.1.2 (GraphPad Software, LCC). P values were considered significant when *P < 0.05 (column statistics *t*-test). Data were obtained in 4 independent experiments.

### PrestoBlueTM Cell Viability assay

PrestoBlue™ Cell Viability reagent (Invitrogen, A13262) was used to assess cell viability. In this assay we used blank (no cells), not treated, mock transfected and siRNA-transfected U87 IDH-WT and IDH-MUT cells. Transfection was carried out as described earlier. Cells were seeded at 24 well plates at 40 k/well. Time points for measuring the viability were 12 h, 24 h, 48 h and 72 h post nucleofection. PrestoBlueTM Cell Viability reagent was added for 30 min incubation in 37℃ (with gentle shaking) at a final concentration of 1x. A portion of PrestoBlue diluted in the medium was kept at the same conditions to be used as a blank. After incubation, 100 ul of medium from each well was transferred to 96 well plate and the fluorescence was read at an excitation/emission wavelength of 560/590 nm using BioTek Synergy HTX fluorimeter. Data was analyzed in GraphPad Prism using Wilcoxon matched-pairs signed rank test, two-tailed. For each timepoint, mock transfected cells viability was normalized to 100%. Data were obtained in 3 independent experiments.

### Matrigel Invasion assay

Matrigel invasion assay was performed using tissue culture inserts (6.5 mm Transwell® with 8.0 μm Pore Polycarbonate Membrane Insert, Corning) coated with the Growth Factor Reduced Matrigel™ Matrix (BD Biosciences, 356,231). 50 μL of the Matrigel™ Matrix (1 mg/mL) diluted in fresh DMEM medium was dried under sterile conditions (37 °C) for 4.5–6 h. The medium was added to the wells 1 h before seeding the glioma cells into inserts. The U87 IDH-WT and IDH-MUT cells were double transfected 54 h prior to plating in matrigel-covered chambers; mock transfected and not transfected cells were used as control. The cells were seeded on matrigel-covered membrane at 45 k per insert in 5% FBS-DMEM. The cultures were placed in a 37 °C humidified incubator with 5% CO_2_. After 18 h, the inserts were washed with PBS, had their inside cleaned with a cotton swab, and were placed in ice-cold methanol for 20 min to fix the cells that had migrated. The membranes were then cut out from the Transwell® inserts and mounted using VECTASHIELD Antifade Mounting Medium with DAPI (Vector Laboratories, H-1800). Cell images were taken within five independent fields (bottom, top, left, right side, and a center) of each specimen, using a fluorescence microscope (Leica DM4000B, 5 × objective). Numbers of migrating cells’ nuclei were counted using ImageJ software. Experiments were performed in duplicates six times in total. Statistical analysis was performed using GraphPad Prism software. The groups were compared using Wilcoxon matched pairs signed rank test and differences considered significant when *P < 0.05.

### REST-Chromatin immunoprecipitation in U87 cells and glioma tumors

U87 IDH-WT and IDH-MUT cells were plated at 10 cm plates, and the following day the cells were trypsinized, centrifuged, resuspended in PBS and fixed with 1% formaldehyde for 10 min. The cells were lysed, and chromatin was isolated and fragmented by sonication (Covaris). Fragmented chromatin was loaded onto REST antibody (Millipore, CS200555) or IgG (Millipore, PP64B)—coated agarose beads (Invitrogen). Immunoprecipitated complexes were eluted, and DNA was purified using DNA Clean&Concentrator kit (Zymo Research). REST IP in tumor cells was performed as previously described [27].

### ChIP sequencing

DNA libraries for chromatin immunoprecipitation sequencing were prepared using QIAseq Ultra Low Input Library Kit (QIAGEN, ZZ-QG-180492) for two independent REST-ChIP experiments. Briefly, DNA was end-repaired, adenosines were added to the 3′ ends of dsDNA and adapters were ligated (adapters from NEB, Ipswich, MA, USA). Following the adapter ligation, uracil was digested by USER enzyme from NEB (Ipswich, MA, USA) in a loop structure of the adapter. Adapters containing DNA fragments were amplified by PCR using NEB starters (Ipswich MA, USA). Library quality evaluation was done with Agilent 2100 Bioanalyzer using the Agilent DNA High Sensitivity chip (Agilent Technologies Ltd, 5067–4626). Quantification and quality evaluation of obtained samples were done using Nanodrop spectrophotometer (Thermo Scientific, NanoDrop products), Quantus fluorometer and Quanti Fluor ONE dsDNA Kit (Promega, E4870) and 2100 Bioanalyzer (Agilent Technologies). The average length of the DNA fragments in the library was 300 bp. The libraries were run in the rapid run flow cell and were single end sequenced (65 bp) on HiSeq 1500 (Illumina).

### RNA sequencing

Total RNA was obtained from 3 passages of each U87 IDH-WT and IDH-MUT that were either not treated, control- or REST-siRNA/KAISO-siRNA transfected as described earlier (Dharmacon, Lonza, and Invitrogen), 500 ng of RNA was used for cDNA synthesis for transcriptome sequencing. mRNA sequencing libraries were prepared using KAPA Stranded mRNAseq Kit (Roche, 07962193001), according to the manufacturer's protocol. Briefly, mRNA molecules were enriched from 500 ng of total RNA using poly-T oligo-attached magnetic beads (Kapa Biosystems). The first-strand cDNA was synthesized using a reverse transcriptase. Second cDNA synthesis was performed to generate double-stranded cDNA (dsDNA). Then, the adapter was ligated, and the loop structure of the adapter was cut by USER enzyme (NEB, Ipswich, MA, USA). Amplification of obtained dsDNA fragments containing the adapters was performed using NEB starters (Ipswich, MA, USA). Quality control of obtained libraries was done using Agilent Bioanalyzer with High Sensitivity DNA Kit (Agilent Technologies, Palo Alto, CA, USA, 5067–4626). Quantification of the libraries was done using Quantus Fluorometer and QuantiFluor Double Stranded DNA System (Promega, Madison, Wisconsin, USA, E4870). The libraries were run in the rapid run flow cell and were paired end sequenced (2 × 76 bp) on HiSeq 1500 (Illumina, San Diego, CA 92122 USA).

### U87 whole genome DNA methylation sequencing

Twelve U87-MG cell line samples were analyzed in total: six IDH-MUT samples, out of which three were siREST and three siCTRL treated. The other six samples were IDH-WT, out of which three were siREST treated and three siCTRL. Genomic DNA was first mechanically fragmented using a Covaris M220 instrument to produce DNA fragments of 350–400 bp. The next steps included repair of the ends of the obtained fragments, adapter ligation, enzymatic conversion of cytosines and amplification of the library using PCR. Basic quality parameters of ready libraries were checked using Agilent Bioanalyzer (fragment length distribution) and Promega Quantus (concentration) devices. They were found to be correct for all samples. The average length of the library was 482 bp (SD = 11), which is within the range defined as an appropriate (470–520 bp) by the kit manufacturer. Next-generation sequencing libraries were prepared using the NEBNext® Enzymatic Methyl-seq Kit (New England Biolabs, cat. no. E7120). Libraries were sequenced using a NovaSeq 6000 device in paired-end sequencing mode 2 × 150 cycles.

### Patient survival analyses

TCGA transcriptomic data (from both the LGG and GBM TCGA transcriptome datasets) were used to conduct four distinct survival analyses including: 1) GBM IDH-WT patients; 2) *IDH1/2*-MUT GBM and LGG patients; 3) patients with all glioma grades regardless of IDH mutation; and 4) LGG IDH-WT patients, in all of which the patients were stratified into high *REST* expression and low *REST* expression groups. Survival and survminer R libraries were used on censored patients data. To support the association between *REST* expression levels and patient survival, Kaplan–Meier estimators and the log-rank test were calculated. Moreover, the Chinese Glioma Genome Atlas (CGGA) dataset was utilized to analyze overall survival curves among patients with WHO grade 4 glioma IDH-WT, distinguishing between those exhibiting high and low *REST* expression. Similarly, this analysis was extended to G4 astrocytomas IDH-MUT, differentiating between high and low REST expression levels.

### U87 cell line IDH-related phenotype cross validation with TCGA glioma data

In order to validate the U87 IDH-related phenotype model, gene expression differences between U87 IDH-WT and its isogenic IDH-MUT cell line were compared to the IDH-related phenotype in the TCGA glioma transcriptomes dataset. First, log2 fold change (log2 FC) values of differentially expressed genes (DEGs) between U87 IDH-WT and U87 IDH-MUT were compared to log2 FC values of DEGs between TCGA LGG IDH-MUT (defined in TCGA as a mutation in *IDH1/2*) and IDH-WT. To validate the relevance of overlap of DEGs with the same direction of change in U87 and TCGA tumors MUT-WT comparisons, a bootstrapping technique (sampling 10,000 times of the same number of genes that were found down- or upregulated in U87 IDH-MUT/WT comparison) was applied. In this comparison only TCGA LGG samples were included because a number of IDH-MUT G4 samples was insufficient. Next, tumor samples from the TCGA repository (WHO grades 2–4, GBM + LGG set) were divided into IDH-WT (n = 86) and IDH-MUT (n = 365). Two differential analyses were carried out: LGG IDH-WT vs LGG IDH-MUT and U87 IDH-WT vs U87 IDH-MUT. REACTOME Pathways enrichment analysis was performed separately for up- (log2 FC > 0) and down-regulated (log FC < 0) DEGs (FDR < 0.01).

### Transcriptomic data from U87 cells

RNA sequencing reads were aligned to the human genome with the STAR algorithm [32], a fast gap-aware mapper. Then, gene counts were obtained by featurecounts [33] using human genome annotations. The counts were imported to R, normalized for gene length and library size and statistical analysis was done by DESeq2 [34] for the following comparisons: IDH-MUT/WT isogenic cell lines, siREST/siCTRL treated IDH-MUT cell line and siREST/siCTRL treated IDH-WT cell line. Gene pathways analysis (KEGG, Gene Ontology, REACTOME) were performed using clusterProfiler R library [35]. Gene Ontology Biological Processes (GO BP) analysis for DEGs arising from REST knockdown in U87 IDH-WT and U87 IDH-MUT was done separately for down- (log2 FC < 0 and adjusted *p*-value < 0.05) and up-regulated (log2 FC > 0 and adjusted *p*-value < 0.05) genes from a pool of IDH-MUT and IDH-WT DEGs.

### U87 REST ChIP-seq data processing

Template amplification and cluster generation were performed using the TruSeq SBS Kit v3 and 50 nucleotides were sequenced with Illumina HiSeq 1500. After quality filtering (average Phred > 30) and removal of duplicates, reads were mapped to the human genome (hg38) with the BWA MEM tool. Samtools view was used to filter best quality reads (-q 20 and -F 256 flags) before peak calling. The peaks were called using the Model-based Analysis of ChIP-Seq (MACS2) algorithm [36] with default parameters; peaks with q-value < 0.01 were considered in the analysis. Peaks were annotated to genes using ChIPseeker [37] and subsequent pathway analysis was performed with clusterProfiler [35]. The raw ChIP-seq data were deposited in the NCBI GEO database: GSE174308. For further analysis, the consensus REST ChIP-seq peaks were generated as follows: the datasets for experiment repeat 1 and 2 were intersected for each U87 IDH-WT and IDH-MUT datasets, separately. The resulting peaks (present in both repeats of the experiment) were then limited to the length of 200 bp (± 100 bp from the peak summit, that was defined as a middle point of the obtained peak) for further TF motifs and DNA methylation analysis. Peaks were centered and limited to 200 bp in order to have a more consistent and homogenous set of observations. The resulting consensus peaks for U87 IDH-WT and IDH-MUT were then intersected again to yield peaks common to both U87 IDH-WT and IDH-MUT, peaks were also subtracted from each other to receive peaks specific to U87 IDH-WT and specific to IDH-MUT.

### Defining REST-activated and REST-repressed genes

The putative REST target genes were defined as those with a REST ChIP-seq peak within a promoter [37] in at least one of the ChIP-seq experiment repeats in either U87 IDH-WT or U87 IDH-MUT. Annotation of REST ChIP-seq peaks to promoter genes was performed with ChIPseeker. REST ChIP-seq peak U87 IDH-WT and IDH-MUT datasets for both sets were pooled. The peak summits were assigned as the middle point of each existing peak, and 200 bp peak sequences were generated covering 100 bp upstream and downstream of the summit. Next, genes defined as REST targets by ChIPseeker based on U87 IDH-WT and U87 IDH-MUT ChIP-seq experiments were correlated (Pearson’s correlation) with REST expression levels in the joined LGG/GBM TCGA transcriptome dataset. Genes that had significant positive correlation of their mRNA level with REST mRNA level (correlation coefficient > 0.15, Bonferroni corrected *p*-value < 0.0001) were defined as REST-activated, while these having significant inverse correlation (correlation coefficient < -0.15, Bonferroni corrected *p*-value < 0.0001) as REST-repressed.

### Intersection of REST knockdown and REST ChIP-seq data

Gene targets of REST defined by REST ChIP-seq (n = 588 activated, n = 981 repressed) were intersected with REST gene targets as defined by REST knockdown. The REST ChIP-seq gene targets that had decreased expression upon REST knockdown were defined as REST activated targets, while those that had increased expression upon REST silencing were defined as REST repressed targets. Separately, Gene Ontology Biological Process (GO BP) analysis was performed for both groups using clusterProfiler and GO BP database.

## Intersection of REST ChIP-seq peaks with ENCODE database

REST ChIP-seq peaks (peak summit ± 100 bp) were submitted to the Enrichr tool (https://maayanlab.cloud/Enrichr/) to predict transcription factors binding based on ENCODE- deposited data.

### A position weight matrix (PWM) analysis

To identify the transcription factors (TF) motifs within the REST ChIP-seq peaks a PWM analysis was performed on two datasets. The first dataset consisted of the sequences stratified according to the *IDH* gene mutation status (WT-specific n = 1007, MUT-specific n = 114, peaks common between both n = 2647) and the second consisted of sequences assigned to REST-activated (n = 588) or REST-repressed genes (n = 981). For both comparisons, the 200 bp peak sequences were used as an input for known motif search using the PWMEnrich Bioconductor R package [R-PWMEnrich] and open-access HOCOMOCO [38] database with 14 additional REST matrices obtained from ENCODE. To identify TF motifs, 10,765 selected promoters active in tumor samples were used as a background (dataset derived from [27]) to proceed lognormal distribution background correction. Only these motifs for which the *p*-value of motif enrichment was lower than 0.001 were selected for further analysis. Due to the notable difference in the number of sequences between IDH-WT (n = 1007) and IDH-MUT (n = 114), an additional step was added for WT/MUT comparison. Namely, ten draws of 114 sequences from the IDH-WT sequence pool were made. A separate motif search using the PWMEnrich R package was performed on each of these ten sets. In parallel, the same analysis was performed on MUT-specific sequences (n = 114). Next, motifs common between the sum of TF motifs from ten draws and the motifs identified on the set of all available sequences were selected. Further analyzes were performed identically for both comparisons (IDH-MUT vs IDH-WT and REST-activated vs REST-repressed). To identify transcription factor binding sites (TFBS), defined as each occurrence of the motif in the sequence, an online FIMO tool [39] from MEME Suite 5.0.1 was used. FIMO scans sequences as double stranded and matches motifs to both forward and backward strands. The *p*-value threshold for the FIMO output was set to 0.001. For further analysis, only the TFBS with the q-value lower than 0.05 were considered (correction done using Benjamini and Hochberg procedure). The TF motifs specific for these TFBS were grouped by sequence using the STAMP website tool (http://www.benoslab.pitt.edu/stamp/). To compare motif sequences during grouping, the Pearson correlation coefficient was used. As an aligning method, the ungapped Smith-Waterman followed by an iterative refinement multiple alignment strategy was used. Finally, the UPGMA tree-building algorithm was used. An enrichment analysis was done using Fisher's exact test with an FDR correction. Transcription factor families were identified based on the HOCOMOCO database, which uses data from the Classification of Transcription Factors in Mammalia database (https://hocomoco11.autosome.ru/human/mono?full=true). The CentriMo tool from MEME Suite 5.4.1 (https://meme-suite.org/meme/tools/centrimo) allowed to determine the position distribution of each motif on the sequences, check the local enrichment of the given sequences with selected motifs, and calculate the static significance of the result using the binomial test. The REST-repressed and REST-activated sequences comparison was performed for the entire sequence length assuming E-value threshold ≤ 10.

### REST peaks hierarchical clustering

To cluster by similarity the peaks with detected REST and KAISO motifs, each peak was changed into a binary vector, where a REST or KAISO motif was assigned value = 1 if present in a peak or value = 0 if absent in a peak. Such vectors were used as the input into hierarchical clustering performed with the Heatmap function from the ComplexHeatmap R package, using the default setting. Grouping was done by rows.

### DiffMeth module for DNA methylation analysis

U87 DNA methylation data were processed with CytoMeth tool to obtain DNA methylation level as beta value (β-value) in a single nucleotide resolution. Next, to identify statistically significant differences in methylation level of the given DNA regions among defined groups of samples a DiffMeth module from CytoMeth tool was used on both U87 and glioma Atlas datasets. The input provided to this module consisted of: (1) DNA regions of interest defined in a standard bed file format (chromosome, starting position, ending position, name of the region, name of the gene, strand); (2) A set of standard bed files resulting from CytoMeth tool, containing single cytosines methylation β-value with their positions on a given chromosome for a given sample. Each sample was defined by a separate bed file; (3) A csv file defining the sample set, which contained methylation file name, sample name/ID, sample group (i.e. various types of glioma groups to be compared), ignore flag, and (4) Diffmeth input parameters defined in yaml file. The first step of the analysis (significance criterion) is based on a standard chi2 statistical test where all groups are compared to each other (pair by pair) to discover possible differences between two groups out of *n* provided. Chi2 test compares distribution of β-values that belong to one of the following ranges: [0.0–0.2), [0.2–0.6), [0.6–1.0] for a given region, two compared groups and CG context. Finally in this step *p*-value is calculated with false discovery rate (FDR) correction. The second step (significance criterion) is based on Kruskal–Wallis statistical test applied on all groups at once similarly as before: for a given region and CG context and *p*-values corrected by FDR. The final interesting differential regions can be selected by intersection of results from both criteria where corrected *p*-value < 0.05.

### DNA methylation data analysis

Complete methylomes of IDH-MUT (siREST, siCTRL) and IDH-WT (siREST, siCTRL) were limited to sequences covered by the REST-ChIPseq peaks (n = 3833). Next, cytosines with a coverage of less than 7 reads were excluded from further analyses. A mean DNA methylation β-value for each cytosine locus was calculated for each sample type (WT-siCTRL, WT-siREST, MUT-siCTRL, MUT-siREST). Then the difference in DNA methylation level as well as fold change was computed between certain groups of samples. The difference in DNA methylation β-value >  = 0.15 was assumed as significant. Chi2 test was used to compare the distribution of β-value of background cytosines (all cytosines within REST-peaks) with differentially methylated cytosines (showing higher or lower methylation pattern in IDH-MUT cell lines) across common, IDH-WT or IDH-MUT specific REST-ChIPseq peaks; results were found significant with p < 0.001.

For both glioma Atlas and U87 cell line datasets, to identify regions with differential DNA methylation the DiffMeth module with FDR correction for multiple testing was used. The results with FDR < 0.05 were considered statistically significant. Differential DNA methylation analysis was performed on the two region types: peaks that cover a sequence of ± 100 bp from the peak summit, and DNA sequence where the presence of a TF motif was statistically confirmed. Importantly, when computing a mean methylation for REST or KAISO motifs, if a cytosine was overlapped by multiple motifs of a single TF (due to motifs overlapping), its β-value was counted once. When a cytosine was within motifs of different TFs (i.e. REST or KAISO), its β-value was counted separately to each TF motif. Following that, for each cytosine, a mean β-value across samples was computed and discretized into low [0–0.2], medium (0.2–0.6] or high (0.6–1]. Low β-values correspond to DNA hypomethylation, whereas high β-values indicate DNA hypermethylation.

### Correlation of gene expression with glioma cell states

Data were analyzed in R, expression of investigated gene was correlated (Pearson’s correlation) with certain cell states: NPC-like1, NPC-like2, MES-like1, MES-like2, OPC-like, AC-like, G1S and G2M as defined by [26].

### Variance analysis in siREST/siKAISO RNAseq data

Counts from siREST and siKAISO experiments were uploaded to R and normalized in DESeq2, then variance was calculated separately for IDH-MUT and IDH-WT samples in siCTRL and siREST or siKAISO samples for each genes in dataset (n of genes = 61,541). Decile values for gene expression variance (values dividing sorted values from lowest to highest in exactly 10 chunks) were calculated separately for IDH-MUT and IDH-WT samples.

## Results

### *REST* expression is positively correlated with glioma malignancy and the presence of the *IDH* mutation

**Fig. 1:**
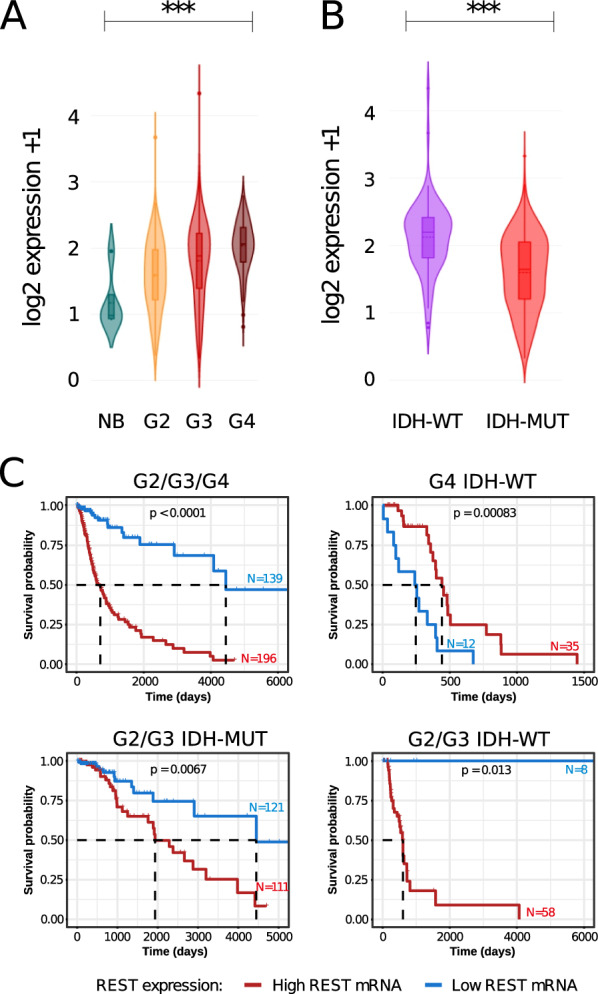
*REST* expression and survival analysis in the glioma TCGA dataset. **A** Violin plots showing expression of *REST* in a TCGA dataset across different glioma grades and normal brain. Significance of difference in gene expression was calculated with the Welch’s ANOVA test; ***p-value < 0.001. **B** Violin plot showing *REST* expression between *IDH1* wild type and *IDH1* mutants in G2/G3 gliomas. Statistical significance calculated with the two-sample Wilcoxon test; ***p-value < 0.001. **C** Kaplan–Meier overall survival curves for the patients with WHO grade 2, 3, and 4 gliomas (top left); IDH-WT G4 glioma (top right); IDH-MUT G2/G3 gliomas (bottom left); IDH-WT G2 and G3 gliomas (bottom right). The patients were divided into high or low *REST* expression groups. The patients alive at the time of the analysis were censored at the time of the last follow-up. Statistical significance was computed using the Log Rank Test

We analyzed *REST* expression in The Cancer Genome Atlas (TCGA) transcriptomic data encompassing normal brain (NB) tissues, LGG (Lower Grade Gliomas, WHO grades 2 and 3) and GBM (glioblastoma, WHO grade 4) samples. The expression of *REST* was the lowest in normal brain tissue and the highest in the WHO grade 4 gliomas, with *REST* expression increasing with glioma malignancy from G2, through G3 to G4 (Fig. 1A). The differences in *REST* expression between NB, G2, G3 and G4 were significant (adjusted *p*-value < 0.001). Interestingly, within the LGGs, *REST* expression was significantly higher in *IDH1/2* mutated (IDH-MUT) gliomas than in wild type samples (IDH-WT) (Fig. 1B; two-sample Wilcoxon test).

Next, we investigated whether *REST* expression had a prognostic value in glioma patients' survival. We found that *REST* expression is a strong negative prognostic factor for patient’s survival in all gliomas (LGG and G4 samples). Patients with low *REST* expression had a favorable prognosis (Fig. 1C, top left). Interestingly, patients with IDH-WT GBMs and high *REST* expression had longer survival (Fig. 1C top right), while patients with IDH-MUT LGGs (Fig. 1C, bottom left) and IDH-WT LGGs with high *REST* expression (Fig. 1C, bottom right) had shorter survival. However, the results of IDH-WT LGG must be taken with caution since the sample size was small due to a scarcity of these tumors.

### Commonality of differentially expressed genes in human IDH-WT and IDH-MUT U87 cells and the TCGA dataset

We took advantage of having isogenic U87 glioma cells with different *IDH1* status and analyzed transcriptomic profiles of U87 IDH-WT and U87 IDH-MUT glioma cells. Despite some controversies regarding the origin of the cells, their transcriptomic profiles are similar to glioblastoma [40]. We found significant differences in gene expression confirming transcriptomic deregulation (Additional File 1A, B). The REACTOME pathway analysis of differentially expressed genes (DEGs) between IDH-WT and IDH-MUT cells revealed a vast number of genes associated with extracellular matrix (ECM) and its reorganization (Additional File 1B).

**Fig. 2:**
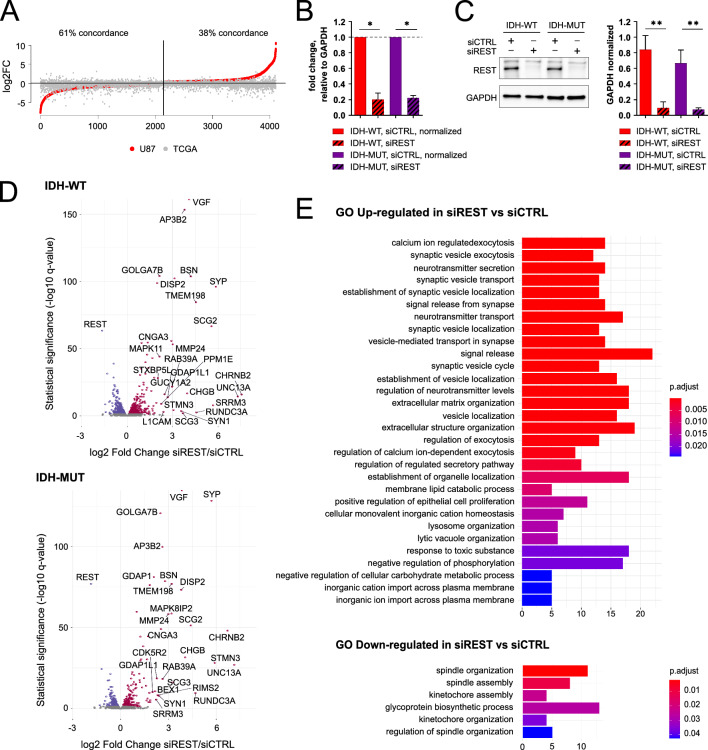
Effects of REST knockdown on gene expression in IDH-WT and IDH-MUT U87 cells. **A** Sorted values of log2 fold change (log2 FC) for DEGs in the IDH-MUT vs IDH-WT comparison in U87 cells are presented as red dots. Values for the same genes inferred from the IDH-MUT vs IDH-WT comparison in TCGA G2/G3 gliomas are overlaid as gray dots. Percentage of log2 FC direction concordance between IDH-MUT vs IDH-WT in U87 cells and glioma tumors was calculated. A number of DEGs is indicated. Black vertical line separates genes with higher expression in IDH-MUT U87 versus IDH-WT U87 from the genes with higher expression in IDH-WT U87 versus IDH-MUT U87. **B** Relative expression of *REST* in IDH-WT and IDH-MUT U87 cells at 72 h of REST silencing with siRNA. mRNA levels in transfected cells were determined with quantitative PCR and normalized to *GAPDH* expression in the same sample. Data are represented as mean ± SEM, n = 4 independent experiments, *p < 0.05, two-tailed Mann–Whitney test (WT: *p* = 0.0286; MUT: *p* = 0.0286). **C** Levels of REST protein in IDH-WT and IDH-MUT U87 cells at 72 h after transfection with control or REST specific siRNAs determined with Western blotting. Immunoblots were analyzed by densitometry. Data are represented as mean ± SEM, n = 4. **p < 0.01 (WT: *p* = 0.0018, MUT: *p* = 0.0052; two-tailed ratio-paired *t*-test). No difference was observed in the level of REST protein between IDH-MUT and IDH-WT controls (siCTRL) (*p* = 0.226). **D** Volcano plots of the genes differentially expressed between siCTRL and siREST-transfected IDH-WT (upper plot) or IDH-MUT (bottom plot) U87 glioma cells. The axes show log2 fold change (x-axis) and -log10 from adjusted q-value (y-axis). **E** Gene Ontology (GO) Biological Processes (BP) analysis was performed on DEGs common in IDH-WT and IDH-MUT U87 cells. The results are presented as bar plots for pathways upregulated (upper panel) and downregulated (bottom panel) in REST depleted cells

Interestingly, DEGs downregulated in U87 IDH-MUT as compared to U87 IDH-WT were highly concordant with DEGs downregulated in IDH-MUT versus IDH-WT LGG tumors from TCGA (61% concordance, Fig. 2A). As none of the bootstrapped results returned such a high concordance, it is unlikely to be random. The overlap of upregulated genes between glioma cells and TCGA tumors was smaller (38%), less than the median overlap (48%) returned by bootstrapping. Also, the REACTOME pathways for DEGs were similar for the downregulated and dissimilar for the upregulated genes (Additional file 1C, D).

### REST knockdown in U87 glioma cells affects numerous biological processes

To find REST dependent genes in glioma cells with different IDH1 status, we performed siRNA mediated knockdown of REST (siREST) in IDH-WT and IDH-MUT U87 glioma cells. A significant reduction in REST mRNA (Fig. 2B) and protein (Fig. 2C) levels was observed after 72 h of REST silencing in both U87 cell lines. *REST* mRNA levels were reduced by 77% in IDH-MUT, and by 82% in IDH-WT (Fig. 2B), while REST protein levels were reduced by 89% in IDH-MUT and by 90% in IDH-WT as compared to control siRNA (siCTRL) transfected cells (Fig. 2C).

We compared transcriptomic profiles between siREST and siCTRL cells and identified 1,234 DEGs in IDH-WT and 629 in IDH-MUT (Fig. 2D). Out of these, 507 were common for IDH-WT and IDH-MUT. The majority of the common DEGs were upregulated (n = 287), whereas 220 DEGs were downregulated, including *REST,* which had the highest log2 fold change (log2 FC) (Fig. 2D). The Gene Ontology Biological Processes (GO BP) functional analysis was performed independently for the up- and downregulated DEGs in siREST cells. The downregulated DEGs were enriched in cell division-related pathways (Fig. 2E, bottom panel), whereas the upregulated genes were enriched in neuronal-specific pathways, as well as pathways associated to endothelial cell proliferation and ECM organization (Fig. 2E, upper panel).

Since the analysis of DEGs in not treated IDH-WT vs IDH-MUT U87 cells and TCGA LGG tumors also indicated ECM-related pathways as differentially regulated, we tested if REST could be a potential modulator of these pathways depending on the IDH status (Additional File 1BC).

### Regulation of gene expression by REST is modified by the *IDH1* mutation

**Fig. 3:**
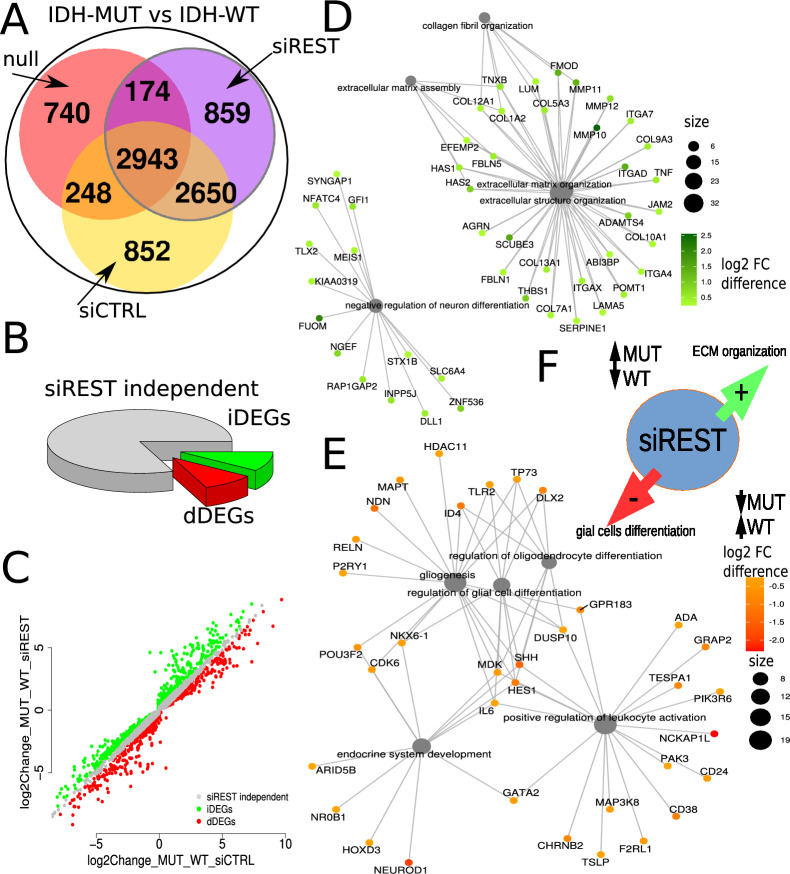
REST knockdown differentially affects expression of genes involved in ECM organization and glial cell differentiation in IDH-WT and IDH-MUT U87 glioma cells. **A** Venn diagram showing the overlap of genes differentially expressed between IDH-WT vs IDH-MUT (IDH-DEGs) in untreated, siREST or siCTRL-transfected U87 glioma cells. Grey circle marks DEGs in the siREST-transfected cells. **B** Subsets of IDH-DEGs dependent (increased FC, red; decreased FC, green) or independent (gray) on REST silencing. **C** Comparison of the fold change difference in gene expression in IDH-MUT or IDH-WT, following siREST/siCTRL transfection. In siREST-transfected cells, a group of DEGs showed a shift in log2 fold change (log2 FC) between IDH-MUT and IDH-WT in siREST compared to siCTRL-transfected cells. Shift of IDH-MUT vs IDH-WT log2 FC in siREST transfected cells when compared to siCTRL transfected cells was either up (green, increased DEGs, iDEGs) or down (red, decreased DEGs, dDEGs). Difference in log2 FC was assumed significant when log2 FC shift between IDH-MUT and IDH-WT comparisons in siREST and siCTRL was > 0.25 and adjusted *p*-value < 0.05. **D** Gene Ontology Biological Processes (GO BP) pathways analysis of the iDEGs, showing genes associated with ECM organization and negative neuronal differentiation. **E** Gene Ontology Biological Processes (GO BP) pathways analysis of the dDEGs, showing genes associated with glial differentiation and immune/endocrine pathways. **F** Graphical summary showing the opposite effect of REST knockdown in IDH-WT and IDH-MUT on the expression of genes related to ECM organization and cell differentiation

To evaluate an impact of REST on gene regulation in the context of *IDH1* mutation, we performed three pairwise comparisons of transcriptomic profiles of IDH-WT and IDH-MUT U87 cells. Differential analysis between IDH-WT vs IDH-MUT was performed on RNA-seq data from the untreated, siCTRL- and siREST-transfected U87 IDH-WT and IDH-MUT cells. DEGs identified in these comparisons were intersected (Fig. 3A) to pinpoint silencing-specific effects. We discovered common 2,943 DEGs (Fig. 3A) showing a strong influence of the IDH-related phenotype on gene expression. To investigate the effect of REST knockdown on IDH-phenotype dependent genes, we focused on genes significantly altered in siREST cells (n = 6,626) (Fig. 3A, delimited in a gray circle) and compared DEGs fold changes in control and siREST cells. DEGs with similar fold changes in siREST and siCTRL comparisons were considered REST independent (Fig. 3B). We defined DEGs as increased DEGs (iDEGs) when they had log2 FC difference between siREST and siCTRL above 0.25 and decreased DEGs (dDEGs) as those that had log2 FC lower than -0.25 (Fig. 3C).

The GO BP pathway analysis demonstrated that iDEGs showed the enrichment in genes involved in ECM organization and negative regulation of neuron differentiation (Fig. 3D). Within these pathways, the highest log2 FC increase was observed in several genes encoding integrins (*ITGAD*) and metalloproteinases (*MMP10*). In contrast, the enrichment for dDEGs showed genes related to reproductive, developmental, and glial differentiation pathways and positive regulation of leukocyte activation (Fig. 3E). Increased expression of genes related to ECM in IDH-MUT siREST cells and their decreased expression in siREST IDH-WT cells, contrasted with decreased expression of glial differentiation-related genes in IDH-MUT siREST cells and their increased expression in IDH-WT siREST cells (Fig. 3F). These findings point to a potential role of REST in a switch between ECM organization and cell differentiation in cells with the different IDH status.

To corroborate these observations, the transcriptomes of G4 primary glioma cell lines were analyzed [30]. Comparison between IDH-MUT (2 astrocytomas G4) and IDH-WT (10 GBMs G4) samples revealed DEGs, which show a considerable overlap with the U87 DEGs dependent on the IDH status only among downregulated genes (Additional File 2A). We demonstrate downregulated ECM-related DEGs from the Fig. 3D in IDH-MUT vs IDH-WT in U87 cells (Additional File 2B) and primary IDH-MUT vs IDH-WT G4 gliomas (Additional File 2C). Out of 18 downregulated ECM-related DEGs in U87 cells 13 genes were similarly regulated in primary cell lines (Additional File 2C), and there is only 1.2% probability of obtaining such a high overlap by chance (bootstrapping procedure, Additional File 2D).

### Invasiveness and expression of genes associated with ECM in REST depleted U87 glioma cells depend on the IDH-related phenotype

**Fig. 4:**
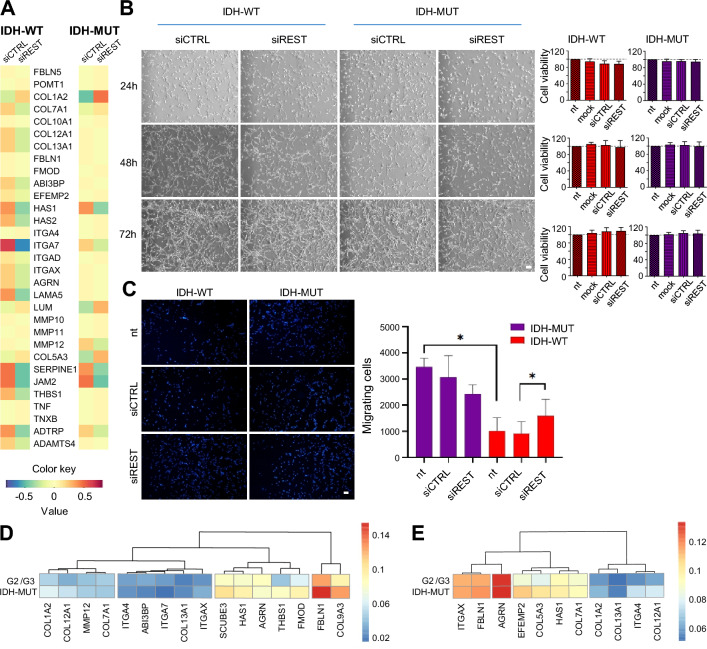
REST knockdown affects invasion of glioma cells and expression of genes implicated in cell migration/invasion. **A** Extracellular matrix organization GO BP-related IDH-DEGs modulated by siREST (selected from functional analysis from Fig. 3C) presented as a heatmap for IDH-WT (left panel) and IDH-MUT (right panel) siREST (right column in each panel) vs. siCTRL (left column in each panel) U87 glioma cell lines. **B** Bright field microscopy images of IDH-WT and IDH-MUT U87 cells 24-, 48- and 72 h after siRNA transfection; scale bar = 200 µm. Cell viability after 24-, 48- or 72 h of REST silencing measured with a PrestoBlue assay. Data are represented as mean ± SEM, n = 3, Wilcoxon matched-pairs signed rank test, two-tailed (*p* > 0.05). Dotted line at 100% denotes a viability of mock-transfected cells. **C** Invasiveness of IDH-WT and IDH-MUT U87 cells measured with a Matrigel invasion assay. The cells were either not treated (nt) or transfected with siCTRL or siREST. The fluorescence microscopy images (scale bar = 200 µm) show representative fields of Matrigel inserts and the bar plot shows quantification of the migrating cells. Data are presented as mean ± SEM, n = 6, *p < 0.05, Wilcoxon matched-pairs signed rank test. IDH-WT siCTRL: 908.8 ± SEM = 460; IDH-WT siREST: 1597 ± SEM = 622.3; not treated IDH-MUT: 3472 ± SEM = 324.7; not treated IDH-WT: 1019 ± SEM = 490.6; *p* = 0.0313. **D** Hierarchical clustering based on mean DNA methylation level within promoters (TSS -2000/ + 500 bps) of ECM genes whose DNA methylation was significantly different (FDR < 0.05) between IDH-MUT and G2/G3 IDH-WT tumor samples deposited in glioma Atlas. **E** Description as in (D) but for genes with significantly differential DNA methylation in gene bodies

The enrichment of biological pathways related to ECM organization and cell differentiation found in REST depleted glioma (genes listed in Fig. 4A), led us to study invasiveness and viability of the cells. Viability of both IDH-WT and IDH-MUT U87 glioma cells was not significantly affected by REST knockdown post-transfection as measured with the PrestoBlue assay (Fig. 4B). The invasion of REST depleted U87 IDH-WT cells, quantified with a Matrigel assay, increased by 75% compared to control cells (Fig. 4C, migrating cells: siCTRL: 908.8 ± SEM = 460; siREST: 1597 ± SEM = 622.3). The opposite, but not statistically significant trend, was observed in U87 IDH-MUT. Invasion of the untreated cells was strongly influenced by their IDH mutation status (Fig. 4C, migrating cells IDH-MUT: 3472 ± SEM = 324.7; IDH-WT: 1019 ± SEM = 490.6).

DEGs whose fold change between U87 IDH-MUT and U87 IDH-WT was increased by REST (iDEGs, Fig. 3D) included a group of genes implicated in the ECM organization pathway (Fig. 4A). To validate if these genes depend on REST, their expression was correlated with *REST* expression in the TCGA glioma dataset (G2/G4). The majority of them significantly correlated with *REST* either in G4 (Additional File 3, left panel), LGG IDH-MUT (Additional File 3, middle panel) or in LGG IDH-WT (Additional File 3, right panel), supporting the notion that REST is required to regulate ECM-related gene expression, but its targets may differ depending on the IDH status. Finally, using tumor methylome data of IDH-WT and IDH-MUT gliomas from the glioma Atlas [27], we found that 16 out of the 32 identified ECM related genes had significantly differential DNA methylation in gene promoters (Fig. 4D) and 11 within gene bodies (Fig. 4E).

### DNA methylation at REST ChIP-seq peaks in REST depleted IDH-WT and IDH-MUT U87 cells

REST knockdown had a modest effect on DNA methylation in the U87 cells for a limited number of cytosines within REST ChIP-seq peaks. Out of 308,816 cytosines tested, there was no difference between IDH-WT siREST and siCTRL samples for over 99% of loci. For 79 loci β-values were lower in IDH-WT siREST than siCTRL (Additional File 4A, values lower than zero) and for 96 were higher (Additional File 4A, values above zero). The largest difference of β-value was 0.08 (Additional File 4A). Similarly, for IDH-MUT, out of 317,501 loci tested, the lower β-values in siREST than siCTRL samples were found in 54 loci and in 81 loci were higher. Here, the highest difference between IDH-MUT siREST and siCTRL β-values was 0.09 (Additional File 4B). Moreover, among differential cytosines, having a difference in DNA methylation β-value >  = 0.15 between DH-WT and IDH-MUT, the pattern of computed differences between IDH-WT and IDH-MUT was fairly constant regardless of REST knockdown (Additional File 4C—comparison of IDH-WT and IDH-MUT transfected with siCTRL; Additional File 4D comparison of IDH-WT and IDH-MUT transfected with siREST). Out of all cytosines (n = 833) assigned as highly methylated in IDH-MUT, only one did not match between siREST and siCTRL samples, while in cytosines lower methylated in IDH-MUT (n = 746) only two did not match between siREST and siCTRL. The observed differences between IDH-MUT and IDH-WT for siCTRL and siREST samples were almost symmetrical (Additional File 4E), suggesting a similar effect of REST knockdown in U87 cells regardless of their *IDH* mutation status.

We discovered that it is more likely that the differential cytosines (difference in DNA methylation β-value >  = 0.15) will appear in IDH-MUT specific or common REST peaks than in IDH-WT specific peaks (Chi2 test *p* < 0.001; see the next chapter for details on peak categorization). Similarly, higher or lower methylation was found more frequently in IDH-MUT specific and common peaks than in IDH-WT peaks (Additional File 4E). Finally, we documented that proportionally to the background, the highest enrichment of differentially methylated cytosines within REST ChIP-seq peaks was found in IDH-MUT specific ones (Additional File 4F).

### Characterization of REST ChIP-seq peaks in IDH-WT and IDH-MUT U87 cells

**Fig. 5:**
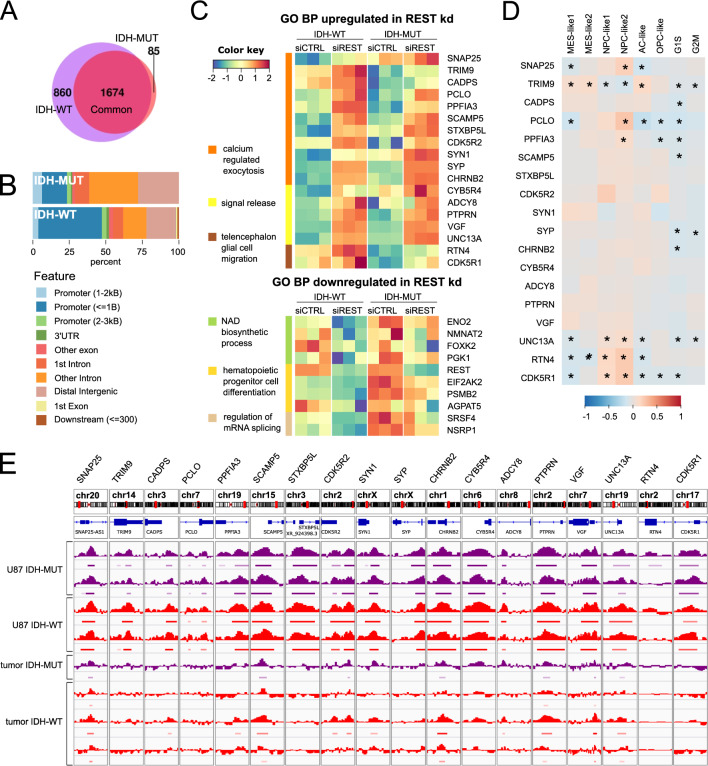
Genes affected by REST knockdown and having REST ChIP-seq peaks belong to migration and differentiation pathways. **A** Intersection of genes assigned to REST ChIP-seq peaks in IDH-WT and IDH-MUT U87 cells. Peaks were assigned to genes following the R ChIPseeker library assignment to the promoter region. **B** Annotation of identified REST ChIP-seq peaks to genomic regions. **C** Upper panel: top GO BP pathways upregulated in primary repressed REST targets were related to calcium release exocytosis, signal release and encephalon glial migration pathways. Lower panel: Main GO BP pathways downregulated in primary activated REST targets were related to: NAD biosynthetic process, hematopoietic progenitor cell differentiation and regulation of mRNA splicing pathways. **D** Expression of genes upregulated in REST depleted cells was correlated with cellular states in single-cell RNAseq data [26]. Pearson correlation was calculated, and a color key scale was used to present its values in range from -1 to 1, correlation significance was marked (*) when adjusted *p* < 0.05. **E** Genomic view from the Integrative Genome Viewer on genes upregulated in REST depleted cells and gliomas; for each sample a histogram of reads was normalized to the input reads (bigwigg file) and bed file from ChIP-seq experiment is shown. REST ChIP-seq data on four U87 glioma cell repetitions (2 × IDH-MUT and 2 × IDH-WT) and 4 tumor samples (1 × IDH-MUT and 3 × IDH-WT) are shown. IDH-MUT samples are color-coded in purple and IDH-WT in red

ChIP-seq was employed to identify REST binding sites in U87 glioma cells. The analysis revealed almost four thousand REST ChIP-seq peaks out of which 2,647 were common in IDH-WT and IDH-MUT cells, while 114 were specific to IDH-MUT and 1,077 to IDH-WT cells. REST ChIP-seq peaks were annotated to genes (hereafter referred to as REST targets). Consistently, the majority of REST targets were shared between IDH-WT and IDH-MUT cells (n = 1674), but 85 genes were specific to IDH-MUT and 860 IDH-WT cells (Fig. 5A). Most of the IDH-MUT specific peaks were located in the intergenic or intronic regions, while the IDH-WT were located mainly in gene promoter regions (Fig. 5B). The lower number of binding sites identified in IDH-MUT (Fig. 5A) and more distal IDH-MUT specific binding sites localization from the transcription start site (Fig. 5B) indicate that the role of REST in IDH-MUT is different than in IDH-WT.

The REACTOME pathway analysis of genes annotated to REST peaks indicated the gene enrichment in ten pathways common to IDH-WT/MUT and eleven pathways specific to either IDH-WT or IDH-MUT REST targets (Additional File 5A). Ten of these pathways were specific to IDH-WT, and they were related to transcription, translation, nonsense mediated decay, voltage gated potassium channels, infectious disease, and heat shock factor 1 activation. The only pathway significantly enriched in targets specific to IDH-MUT was “Interaction between L1 and ankyrins” (Additional File 5A).

Next, we analyzed potential TFs binding within sequences of REST ChIP-seq peaks assigned to genes specific to IDH-WT (n = 860), IDH-MUT (n = 85), or common to both cell types (n = 1,674) (Fig. 5A). Using EnrichR, we found that ENCODE ChIP-seq peaks of KAISO, known also as ZBTB33 (Zinc Finger and BTB Domain Containing 33), had the strongest enrichment within the REST ChIP-seq peaks specific for IDH-WT cells (Additional File 5C, middle panel). Contrary, in IDH-MUT specific REST ChIP-seq peaks, only ENCODE REST peaks were identified (Additional File 5C, middle panel), while in the REST ChIPseq peaks common to IDH-WT and IDH-MUT, both ENCODE REST and ENCODE KAISO peaks were enriched (Additional File 5C, top panel).

To identify specific TF motifs, present within the REST ChIP-seq peaks specific for IDH-WT or IDH-MUT, or common for both IDH-WT and IDH-MUT, we investigated the peak sequences using all available position weighted matrices (PWMs) deposited in the **HO**mo sapiens **CO**mprehensive **MO**del **CO**llection (HOCOMOCO) database (version 11) and additional 14 REST PWMs from the ENCODE dataset. Using the R PWMEnrich package and the FIMO tool [39], we identified 120 distinct TF motifs within the REST ChIP-seq peaks common to IDH-WT and IDH-MUT, 70 motifs in IDH-WT specific peaks and 14 motifs within the IDH-MUT specific peaks (Additional File 5B). Ten motifs were shared among the three peak types (Additional File 5B). Focusing on KAISO motifs, we discovered that all the three HOCOMOCO motif variants (0.A, 1.A and 2.A) were present within the common and the IDH-WT specific REST ChIP-seq peaks, but none of these motifs were present in the IDH-MUT specific peaks (Additional File 5D).

Finally, we hypothesized that changes in DNA methylation resulting from an IDH status may affect REST binding to its target sites. Therefore, we examined DNA methylation β-values in IDH-MUT, G2/G3 IDH-WT and G4 IDH-WT glioma Atlas samples [27]. Pairwise comparisons of DNA methylation within the sequences assigned as common, IDH-WT or IDH-MUT specific based on REST ChiP-seq peaks in U87 cells were performed separately for different glioma groups (Additional File 6A-C). A similar percentage of common (8%), IDH-WT (6.9%) and IDH-MUT specific (5.5%) peaks was differentially methylated between the three aforementioned glioma tumor groups. In each REST peak type (common, WT-specific, MUT-specific), the largest number of differentially methylated peaks was detected between IDH-MUT and G4 IDH-WT samples (n = 197, 6, 71, respectively; Additional File 6A-C, a middle column). Next, to identify the effect of IDH-related phenotype, we focused on the peaks differentially methylated between G2/G3 IDH-WT and IDH-MUT samples (128 common, 4 WT-specific, 32 MUT-specific; Additional File 6A-C, a left column). Majority of these peaks (n = 135; 82.3%) had higher DNA methylation in IDH-MUT samples (Additional File 6D-F), confirming that the IDH status has a dominant impact on differential DNA methylation of the REST peaks.

### REST-regulated genes in glioma cells belong to cell migration and differentiation pathways

REST knockdown may affect genes that are activated or repressed by REST binding either directly (hereafter referred to as primary REST targets) or indirectly. REST knockdown may also have indirect effects related to altered interactions between REST and other proteins and/or downstream regulatory cascades controlled by primary REST targets (hereafter REST secondary targets). The genes whose expression was significantly changed after REST knockdown and had REST ChIP-seq peaks, were assigned as primary REST targets. GO Biological Pathways enriched among primary repressed REST targets (upregulated after siREST) comprised pathways related to neuronal transmission (signal release, calcium regulated exocytosis) and glial cell migration (Fig. 5C, an upper panel). The pathways related to primary activated REST targets (downregulated after siREST) included NAD biosynthetic process, regulation of mRNA splicing and hematopoietic progenitor cell differentiation (Fig. 5C, a lower panel).

Further, a correlation of REST and its primary targets’ expression was calculated in the TCGA dataset. The direction and strength of the correlations were compared to the direction of DEGs expression fold change after siREST. Majority of the primary repressed REST targets were enriched in the signal release, calcium regulated exocytosis and telencephalon glial cell migration biological pathways (Fig. 5C), and had negative correlation with *REST* expression in G4 TCGA dataset (Additional File 7A). Nearly half of the primary activated REST targets related to NAD biosynthetic process, regulation of mRNA splicing and hematopoietic progenitor cell differentiation (Fig. 5C), had positive correlation with *REST* expression in G4 TCGA dataset (Additional File 7B). Additionally, most of the primary repressed REST targets were consistently downregulated in gliomas compared to normal brain samples, with expression decreasing from G2 and G3 to G4 (Additional File 7C). Primary activated REST targets did not show this pattern in TCGA gliomas (not shown). A number of primary repressed REST targets correlated significantly with glioma cellular states described by [26] (Fig. 5D). Among those, many primary repressed REST targets showed significant positive correlation with NPC-like states and negative correlation with G1S and G2M states, suggesting rather non-cycling cells (Fig. 5D). Contrary to the primary activated REST targets (not shown), the primary repressed REST targets showed concordance of their REST binding sites with those detected in glioma tumors (Fig. 5E).

### Occurrence of TF motifs within the REST ChIP-seq peaks among REST-activated or REST-repressed genes

Most REST-repressed genes were enriched in the GO biological pathways related to neuronal functions, confirming its canonical role as a repressor of neuronal genes in non-neuronal cells (Additional File 8A, left panel). On the other hand, pathways related to the REST-activated targets were more diverse (Additional File 8A, right panel).

**Fig. 6:**
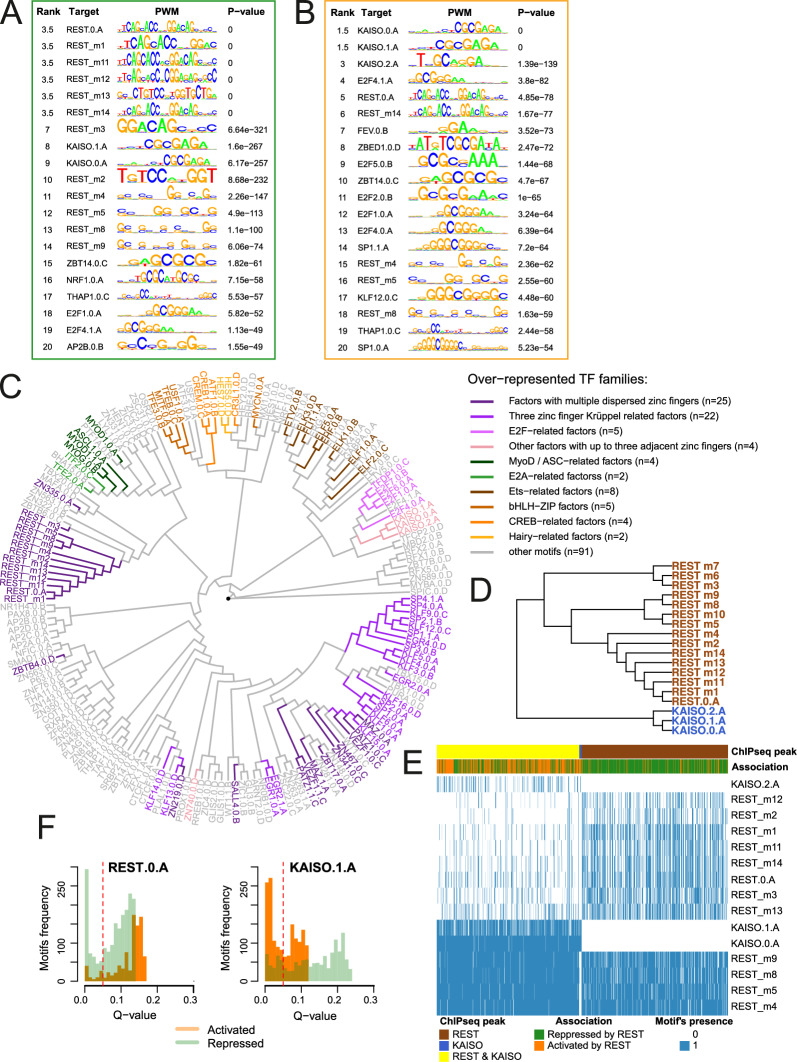
Characterization of REST ChIP-seq peaks and their target genes. **A** and **B** Ranking of the TOP 20 TF motifs identified in the sequences of the REST ChIP-seq peaks assigned to genes repressed by REST (**A**) or activated by REST (**B**). Briefly, *REST* expression was correlated with the genes to which REST ChIP-seq peaks were assigned using TCGA dataset (data from Fig. 5A). Based on the correlation results between *REST* gene expression and REST targets, the genes were divided into repressed or activated by REST. If correlation was statistically significant (adjusted *p* value < 0.05) and correlation coefficient was positive, a gene was assigned as activated by REST, while coefficient was negative, a gene was assigned as repressed by REST. **C** Hierarchical tree of TF motifs for enriched TF families based on PWMs. Shades of green represent motifs from TF protein families overrepresented in REST ChIP-seq peaks unique for repressed REST targets; orange-activated; magenta-motifs overrepresented in the group of motifs present in both repressed and activated REST targets. **D** REST and KAISO (ZBTB33) motifs clustering based on PWMs. **E** Hierarchical clustering of REST peaks according to the identified KAISO and REST motifs. Color-coded bars show the association of a REST peak and its target gene, impact on gene expression (repressed or activated by REST) and the presence of REST and/or KAISO motifs. **F** Q-value and frequency relations for selected KAISO (ZBTB33) and REST motifs within REST-ChIP-seq peaks assigned to genes activated or repressed by REST. To highlight the pattern, bar plots show the full distribution of q-values with a red dashed line indicating significance cut-off point

To determine the exact locations of the TF motifs detected with PWMEnrich in peaks associated with REST-activated and REST-repressed genes, we used the FIMO tool and identified 145 motifs for 119 TFs within the REST peaks assigned to REST-repressed genes and 140 motifs for 115 TFs within the peaks assigned to REST-activated genes (q-value ≤ 0.05). Hierarchical clustering tree of the identified TF motif PWMs revealed well pronounced separation of motifs assigned to the REST-repressed and REST-activated targets (Additional File 8B). The same tree, exhibiting TF motifs sequence similarities, was used to visualize protein families of TFs detected within REST peaks paired with REST-activated or repressed genes (Fig. 6C). The motifs identified in both REST-repressed and activated targets included E2F transcription factor family motifs, which is a classic TF involved in glioma progression [41, 42].

Among the motifs enriched in REST ChIP-seq peaks of REST-repressed genes, were targets for ASCL1, MyoD and E2A-related factors that are engaged in cell differentiation and proliferation, including neuronal (ASCL1) and oligodendrocyte (E2A) differentiation [43], mesenchymal cell proliferation (MyoD) and growth inhibition [44]. In cancer, these TFs act as activators (ASCL1) or inhibitors of the cell cycle progression (E2A) [45]. Motifs overrepresented in REST-activated genes were targets for CREB, Ets family proteins, bHLH-ZIP, and Hairy-related factors. Among others, CREB regulates transcription of the genes coding for a proto-oncogene c-Fos [46] and neuropeptide VGF [47]. The latter was one of the stronger upregulated genes upon REST knockdown (Fig. 2D). Ets family proteins are activated by Ras-MAP kinase signaling pathway and have been implicated in tissue differentiation and cancer progression [48]. Hairy-related proteins typically function as DNA-binding transcriptional repressors that have been shown to inhibit Notch activated *a-actin* [49] and control differentiation [50]. Overrepresentation of the motifs for these TFs in the vicinity of REST binding sites in REST-repressed genes shows a potential contribution of their pathways to the diverse REST effects and its impact on tumorigenesis and patient survival.

To identify the most prevalent TF motifs within the REST peaks associated with REST-activated or REST-repressed genes, we performed TF motif scans using PWMs. The top of the ranking of the highest scoring motifs for the REST-repressed genes contained mainly REST motifs, as expected (Fig. 6A). However, in the ranking of TF motifs in REST-activated genes, the first three positions were occupied by KAISO (Fig. 6B). TF binding motif sequence logos generated for both KAISO and REST PWMs (Additional File 8F, G) and hierarchical clustering of all discovered PWM sequences (Fig. 6C) as well as REST and KAISO PWMs alone (Fig. 6D) showed dissimilarity between these motifs, hence their co-occurrence within the REST ChIP-seq peaks was not due to the PWMs similarity. However, based on the distribution of the statistical significance of the occurrence of TF motifs, we may assume that KAISO motifs are more frequent in the promoters of REST-activated than REST-repressed genes, whereas REST motifs display a somewhat reverse pattern (Fig. 6F). The results of the CentriMo analysis showing the probability of REST or KAISO motif presence in the gene promoters corroborate this observation, as we found more frequent occurrence of KAISO motifs in genes activated by REST (Additional File 8C) than in the genes repressed by REST (Additional File 8D). For the REST motifs in the REST-repressed genes the distribution was similar to KAISO motifs (Additional File 8D, E) while for the REST-activated genes the probability was below statistical significance.

To determine a degree of REST ChIP-seq peaks similarity according to the detected REST and KAISO motifs, each peak was represented by a binary vector where a given REST or KAISO motif was present (value = 1) or absent (value = 0), and a hierarchical clustering on these vectors was performed (Fig. 6E). In general, two clusters of peaks were discovered: 1) represented mostly by REST and KAISO motifs and associated with gene activation; 2) represented only by REST motifs and mostly associated with gene repression (Fig. 6E). Among the reported REST peaks (n = 1,523), 63% were related to repressed and 37% to activated targets. In the majority of peaks related to REST-repressed genes (69.5%) only the REST motifs were found. In contrast, in the majority of peaks related to activated genes (81%) both REST and KAISO motifs were present. Our results imply that KAISO motif occurrence or binding may be an important factor in REST-driven transcriptional activation, but not in gene repression.

### Effect of KAISO silencing on REST-regulated genes

**Fig. 7:**
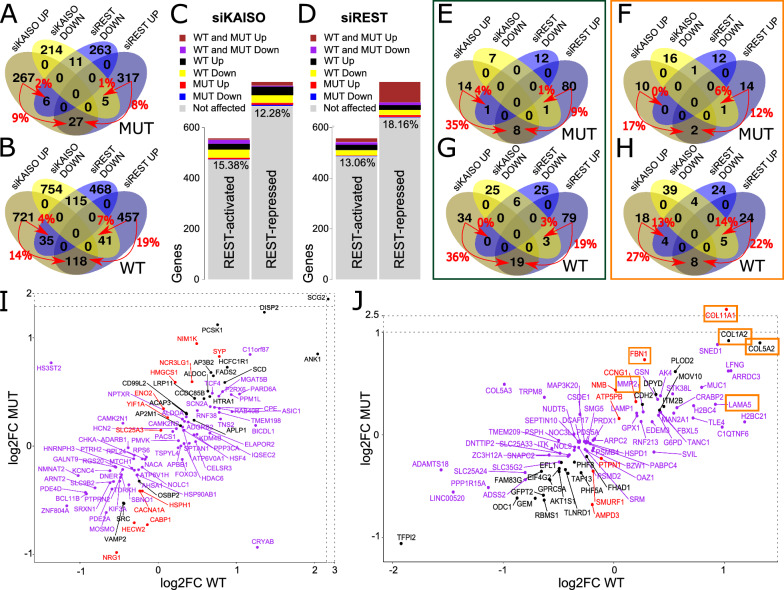
KAISO silencing affects REST-regulated genes. **A** Venn diagrams showing genes: 1) significantly upregulated by siKAISO; 2) significantly downregulated by siKAISO; 3) significantly upregulated by siREST; 4) significantly downregulated by siREST in IDH-MUT glioma cells. **B** Venn diagrams showing genes: 1) significantly upregulated by siKAISO; 2) significantly downregulated by siKAISO; 3) significantly upregulated by siREST; 4) significantly downregulated by siREST in U87 IDH-WT glioma cells. (**C**) Effect of KAISO silencing on gene expression in IDH-MUT and IDH-WT U87 glioma cells. Chi2 analysis between differentially expressed genes categories is presented in Additional File 11. **D** Effect of REST silencing on gene expression in IDH-MUT and IDH-WT U87 glioma cell lines. Chi2 analysis between differentially expressed genes categories is presented in Additional File 11. **E** Venn diagram of genes: 1) significantly upregulated by siKAISO; 2) significantly downregulated by siKAISO; 3) significantly upregulated by siREST; 4) significantly downregulated by siREST in U87 IDH-MUT glioma cells within genes from REST-repressed category. **F** Venn diagrams showing genes: 1) significantly upregulated by siKAISO; 2) significantly downregulated by siKAISO; 3) significantly upregulated by siREST; 4) significantly downregulated by siREST in U87 IDH-MUT glioma cells within genes from REST-activated category. **G** Venn diagrams showing genes: 1) significantly upregulated by siKAISO; 2) significantly downregulated by siKAISO; 3) significantly upregulated by siREST; 4) significantly downregulated by siREST in U87 IDH-WT glioma cells within genes from REST-repressed category. **H** Venn diagrams showing genes: 1) significantly upregulated by siKAISO; 2) significantly downregulated by siKAISO; 3) significantly upregulated by siREST; 4) significantly downregulated by siREST in U87 IDH-WT glioma cells within genes from REST-activated category. **I** Scatter plot of REST-repressed genes with significantly changed expression upon KAISO silencing in IDH-MUT and IDH-WT glioma cell lines. Log2 fold change in gene expression between siKAISO and siCTRL is plotted for IDH-MUT (y-axis) and IDH-WT(x-axis). The genes that had significantly changed expression in both IDH-WT and IDH-MUT are shown in black, the genes specific to IDH-MUT are shown in red, and the genes specific to IDH-WT are shown in purple. **J** Scatter plot of REST-activated genes with significantly changed expression upon by KAISO silencing in IDH-MUT and IDH-WT glioma cell lines. Log2 fold change in gene expression between siKAISO and /siCTRL is plotted for IDH-MUT (y-axis) and IDH-WT (x-axis). The genes that had significantly changed expression in both IDH-WT and IDH-MUT are shown in black, the genes specific to IDH-MUT are shown in red, and the genes specific to IDH-WT are shown in purple. Genes assigned to ECM organization by Gene Ontology Biological Process analysis are framed in orange

We performed KAISO silencing in U87 cells (Additional File 9) to determine a potential overlap between REST and KAISO gene targets in glioma cells. KAISO knockdown was effective as shown by decreased *ZBTB33* mRNA level (Additional File 9A) and effects on known KAISO targets (Additional File 9C). We can’t confirm knockdown at a protein level (Additional File 9B), most likely due to poor antibody specificity. The genes significantly (adjusted *p*-value < 0.05) up- or downregulated by either siREST or siKAISO in U87 cells were independently intersected within IDH-MUT (siKAISO: 481 genes, siREST: 580 genes, Fig. 7A) and within IDH-WT cells (siKAISO: 1475 genes, siREST: 925 genes, Fig. 7B). Interestingly, while in IDH-MUT cells the upregulated genes were almost 3 times more likely to overlap between siREST and siKAISO than the downregulated genes (11 down- and 27 upregulated genes, Fig. 7A), in IDH-WT the numbers of overlapped genes were similar (115 down- and 118 upregulated genes, Fig. 7B). Next, we verified the frequency of REST, KAISO or both TFs motifs within the REST ChIP-seq peaks annotated to genes up- or downregulated in IDH-WT/IDH-MUT after siKAISO. In IDH-WT genes downregulated after siKAISO, REST peaks with both REST and KAISO motifs had significantly higher frequency than REST peaks with only REST motifs. Genes upregulated had more frequent REST peaks with only REST motifs (Chi2 = 8.2, p < 0.01). In IDH-MUT the motifs composition within REST ChiP-seq peaks of up- or downregulated genes after siKAISO did not show significant differences. Moreover, to illustrate similarities or differences between REST and KAISO knockdown, we intersected the sets of DEGs affected by REST or KAISO knockdown with TCGA-based and REST ChIP-seq based (details in Materials and Methods) REST-activated and REST-repressed gene sets (Fig. 7C, D). KAISO depletion affected the expression of 15.38% of REST-activated and 12.28% of REST-repressed genes (Fig. 7C), compared to 13.06% of REST-activated and 18.16% of REST-repressed genes REST depleted cells (Fig. 7D). The observed enrichment in the number of overlapping genes was significant for siKAISO and REST-activated, but not REST-repressed genes (bootstrapping: Additional file 10A, B; chi2 test: Additional file 11) and highly significant for siREST and REST-repressed genes (bootstrapping: Additional file 10A, B; chi2 test: Additional file 11). Next, we checked how many of siREST and siKAISO differentially up- or downregulated genes fall into REST-repressed (Fig. 7E for IDH-MUT, Fig. 7G for IDH-WT) or REST-activated categories (Fig. 7F for IDH-MUT, Fig. 7H for IDH-WT). Compared to previous global analysis (Fig. 7A, B), there was a much greater concordance of siKAISO and siREST upregulated genes in both IDH-MUT (35%, compared to 9%, Fig. 7A, E) and in IDH-WT (36% compared to 14%, Fig. 7B, G) for a subset of genes within the REST-repressed category. On the other hand, the number of genes that overlap between up- and downregulated categories in IDH-WT samples was strikingly reduced (6 down-, 19 upregulated, Fig. 7G), an effect that was not so pronounced in IDH-MUT (0 down-, 8 upregulated, Fig. 7E), possibly due to a lower number of genes considered in general. The situation is very different for REST-activated genes (Fig. 7F, H). While higher proportion of upregulated than downregulated genes overlaps in siREST and siKAISO datasets (4 vs 8 IDH-WT, Fig. 7H, 2 vs. 1 IDH-MUT, Fig. 7F), there is a much higher proportion of genes that have the opposite regulation in siREST and siKAISO in IDH-WT (Fig. 7H). These results suggest a certain degree of synergy between REST and KAISO transcription factors in the regulation of REST-repressed genes, and rather a competition between these TFs in the regulation of REST-activated genes.

Finally, to have an overview of KAISO targets overlapping with REST-activated and REST-repressed genes in glioma, log2 fold change in gene expression between siKAISO and siCTRL for IDH-MUT and IDH-WT were plotted for REST-repressed (Fig. 7I) and REST-activated genes (Fig. 7J). The group of REST-repressed genes affected by siKAISO (Fig. 7I) was heterogeneous. The Gene Ontology Biological Process did not show any significant enrichment for the downregulated genes in this group. In contrast, the upregulated genes showed the enrichment in glycolysis (REST-repressed, data not shown) and a strong enrichment in the ECM organization pathway in siKAISO-affected REST-activated genes (Fig. 7J, Additional file 12).

In many analyses (including siREST DEGs, siKAISO DEGs, and REST-ChIPseq), a smaller number of targets or lower *p*-values were detected in IDH-MUT than in IDH-WT datasets (Additional file 10C, D), that is also in line with a lower number of detected ChIP-seq peaks (Fig. 5A). This suggests deregulation caused by the IDH-phenotype. Indeed, variance in both siREST and siKAISO datasets was always higher in IDH-MUT samples than in IDH-WT (Additional file 10E, F), which may explain lower p-values in most of the analyses.

### Distinct DNA methylation within REST and KAISO motifs of activated and repressed REST targets

**Fig. 8:**
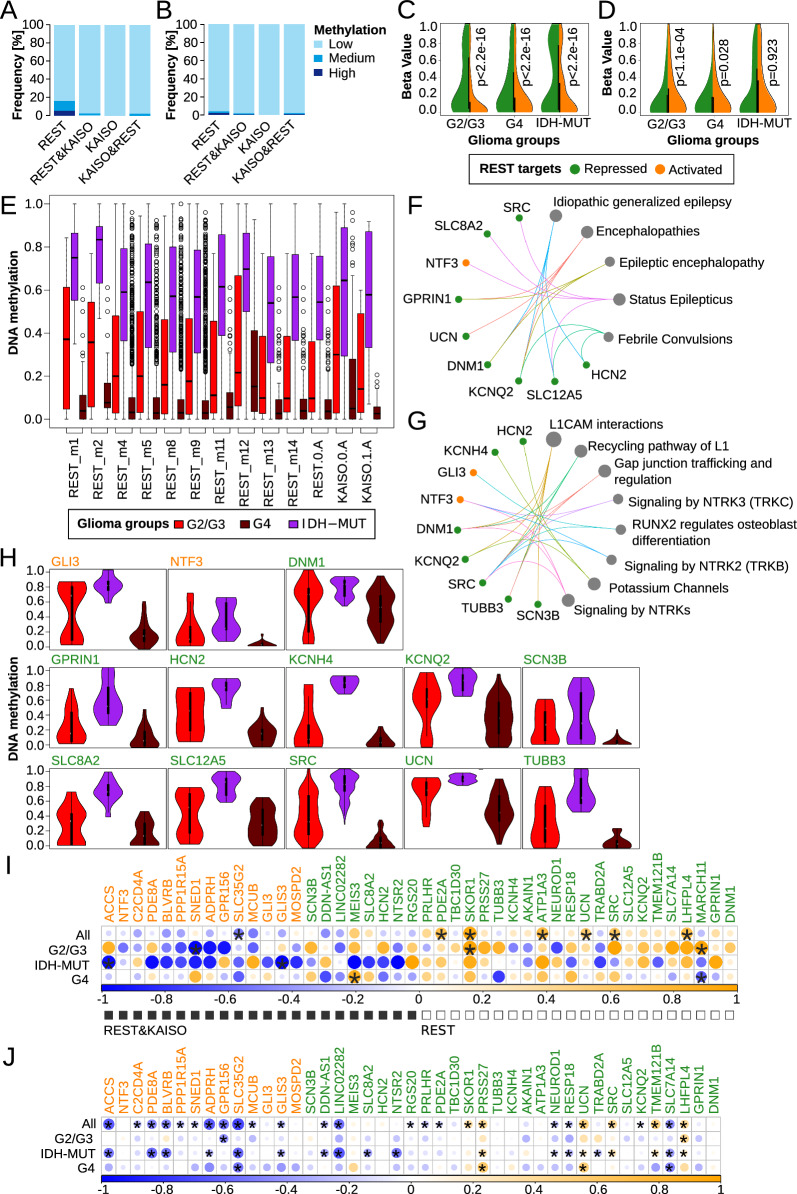
DNA methylation at the selected REST ChIP-seq peaks and its influence on REST targets. **A** DNA methylation of REST or KAISO motifs within REST ChIP-seq peaks assigned to REST-repressed targets in IDH-MUT, G2/G3 IDH-WT and G4 IDH-WT tumor samples from glioma Atlas. Description represents: REST – methylation of REST motifs of peaks lacking KAISO; REST&KAISO – methylation of REST motifs of peaks containing both REST and KAISO motifs; KAISO – methylation of KAISO motifs present in REST ChIP-seq peaks with identified KAISO but not REST motif; KAISO&REST – methylation of KAISO motifs of peaks containing both REST and KAISO motifs. **B** DNA methylation of REST or KAISO motifs within the REST ChIP-seq peaks assigned to REST-activated targets in IDH-MUT, G2/G3 IDH-WT and G4 IDH-WT tumor samples from glioma Atlas. Description as in **A**. **C** Distribution of REST motif DNA methylation in the REST ChIP-seq peaks containing only REST motifs and assigned to the repressed or activated REST targets based on glioma Atlas. **D** Distribution of REST motif DNA methylation in the REST ChIP-seq peaks containing both REST and KAISO motifs and assigned to the repressed or activated targets based on the glioma Atlas. **E** Cumulative distribution of DNA methylation sites (n = 601) in individual motifs. There are 601 sites containing REST or KAISO motifs with significantly different methylation among gliomas from the glioma Atlas. **F** Human disease pathways enriched among 47 REST targets. REST ChIP-seq peaks assigned to these targets had at least one differentially methylated REST or KAISO motif. **G** Description as in (F) but for REACTOME pathways. **H** Distribution of DNA methylation (glioma Atlas) of REST or KAISO motifs within REST ChIP-seq peaks assigned to the genes present in enriched pathways shown in **F** and **G**. **I** Correlation between expression of a REST-target gene and mean DNA methylation of a motif assigned to its promoter. Correlations were performed using the glioma Atlas dataset. Black squares mark REST-targets having REST and KAISO motifs in the REST ChIP-seq peaks, while white squares mark those in which only REST motifs were detected. **J** Correlation between expression of a REST-target gene and mean DNA methylation of its promoter in the TCGA dataset

While in the previous analysis (Additional File 6) we verified DNA methylation of the whole 200 bp sequences of REST ChIP-seq peaks, here we focused on DNA methylation of identified REST and KAISO motifs within the REST ChIP-seq peaks assigned to REST-repressed or activated targets. We studied cytosine methylation in the CpG context with a minimal coverage of 10 reads per cytosine, in the IDH-MUT, G2/G3 IDH-WT and G4 IDH-WT glioma methylomes, deposited in glioma Atlas [27]. These methylomes, covering millions of sites at 1 bp resolution, were intersected with coordinates of KAISO and REST motifs determined within the U87 REST ChIP-seq peaks, resulting in a set of 23,614 unique cytosines. The majority of cytosines occurred within either REST or KAISO motifs, but 2,588 were located within sites overlapped by REST and KAISO motifs. REST motifs located in the REST ChIP-seq peaks lacking KAISO motifs within the same peak and assigned to the repressed REST targets, were significantly enriched (p < 2.2 × 10^–16^) in medium- and hypermethylated cytosines (Fig. 8A, Additional File 7). In these REST motifs, the percentage of hypermethylated cytosines (5.54%) was almost three times higher than hypermethylated cytosines (1.98%) within REST motifs from REST ChIP-seq peaks assigned to the REST-activated targets (Fig. 8A-B, Additional File 13).

Since a vast majority of cytosines were hypomethylated (Fig. 8A-B), methylation variance across IDH-MUT, G2/G3 IDH-WT and G4 IDH-WT gliomas was computed for each cytosine within the REST and KAISO motifs. The obtained variance ranged from 0 to 0.05 among sites, with an average β-value of 0.0067. To explore the methylation pattern within REST motifs, we selected cytosines with variance higher than the average. We found that DNA methylation of REST motifs located within REST ChIP-seq peaks lacking KAISO motifs was significantly higher in peaks assigned to REST-repressed targets than in peaks assigned to REST-activated targets across all glioma groups (Fig. 8C). DNA methylation within REST motifs of REST ChIP-seq peaks containing both KAISO and REST motifs showed significantly higher DNA methylation in peaks assigned to repressed REST targets only in G4 IDH-WT gliomas (Fig. 8D). In G2/G3 IDH-WT samples the methylation pattern was the opposite, and in IDH-MUT no significant differences were detected (Fig. 8D).

The number of sites with predicted REST and/or KAISO motifs within a single REST-peak was high, which did not allow for unequivocal determination which of predicted motifs was actually used for TF binding. Thus, we studied DNA methylation patterns within individual motifs for the REST or KAISO TFs across gliomas using the DiffMeth tool and methylomes of IDH-MUT, G2/G3 IDH-WT and G4 IDH-WT gliomas from the glioma Atlas [27]. Initially, almost 70,000 REST and KAISO motif sites were found within REST ChIP-seq peaks in glioma cells (Additional File 14), and 601 of them were differentially methylated among gliomas, which accounts for 0.9% of the all identified REST and KAISO motif sites (Fig. 8E). The proportion between all predicted sites of individual motifs compared to a number of differentially methylated sites was significantly different (*p*-value < 0.01, Additional File 8). We identified six of the twelve REST motifs as having differential DNA methylation in gliomas (Additional File 14) and a majority of differentially methylated sites of REST motifs were found within REST ChIP-seq peaks corresponding to REST-repressed targets (Chi2 = 97.069, df = 13, *p*-value = 6.119e−15).

Differentially methylated sites (n = 601) among gliomas had the highest median β-value in IDH-MUT and the lowest in G4 IDH-WT gliomas (Fig. 8E). Frequently, more than one motif showed significantly differential methylation in a single REST ChIP-seq peak. We found that sites differentially methylated in gliomas appeared within 47 REST ChIP-seq peaks, out of which 32 were associated with REST-repressed targets, while 15 with REST-activated targets. A complete list of REST targets paired with the REST ChIP-seq peaks containing differentially methylated REST or KAISO motif sites is presented in Additional File 15. For these genes, we searched for associations with diseases using the DisGeNET platform and enrichment of specific biological pathways using the REACTOME database. Nine out of 47 genes were linked to disorders, including epilepsy, encephalopathies, and febrile convulsions (Fig. 8F). Nine genes were associated with significantly enriched REACTOME pathways (Fig. 8G). The largest number of genes (n = 5), all under REST repression, were linked with L1 cell adhesion molecule (L1CAM) interactions. Several genes (n = 6), all assigned as REST-repressed targets, were linked to another three pathways: recycling pathway of L1, gap junction trafficking and regulation, and potassium channels (Fig. 8G). We found that the biological functions associated with L1CAM interactions and cell differentiation (*RUNX2* regulates osteoblast differentiation) are similar to pathways detected to be altered in IDH-WT and IDH-MUT REST depleted cells (Fig. 3). Some of the genes defined as REST-activated targets (i.e. *NTF3* and *GLI3*) containing KAISO motifs within the REST ChIP-seq peaks had a methylation pattern similar to the repressed REST targets across glioma tumor types (Fig. 8H).

To verify whether differentially methylated motif sites affect expression of REST targets, we calculated correlation between mean methylation and gene expression using the glioma Atlas (Fig. 8I) and TCGA datasets (Fig. 8J). The methylome data in the glioma Atlas are dense, thus we were able to calculate TF motif site mean methylation and correlate it with REST target expression. Due to a low number of cytosines covered in the TCGA, mean methylation was computed for the entire promoters. Expression of most REST activated targets negatively correlated with site/promoter methylation levels. Interestingly, for the repressed genes, the positive correlation was more frequently detected (Fig. 8I, J). Exploration of the glioma Atlas data demonstrates that a negative correlation coefficient corresponds well with the presence of the KAISO motif in a ChIP-seq peak assigned to a gene (Fig. 8I black squares) and a negative correlation with its absence (Fig. 8I white squares).

## Discussion

We performed a comprehensive study to identify transcriptional targets of REST and unravel REST regulatory networks in U87 glioma cells with a different IDH status. These findings were validated in lower and high grade gliomas in own and public TCGA datasets. Using a large spectrum of computational methods, ChIP-seq, RNA-seq, DNA methylation and RNAi mediated REST knockdown in U87 cells with a wild type or mutant *IDH1*, we addressed a complex role of REST in gliomagenesis. Recognition of the importance of IDH1/2 mutations in progression of diffuse gliomas advanced our understanding of glioma biology, however the full impact of a state of DNA and histone hypermethylation on gene regulatory networks and cell functions is less clear. Our study demonstrates that REST-regulated gene networks in gliomas are dependent on the *IDH* mutation status, which determines a selection of REST dependent genes involved in ECM organization, glioma invasion and cell differentiation. We uncovered a putative cooperation between REST and KAISO in determining repression or activation of REST targets. Our results point to REST as a valid target in anti-glioma therapy.

The *IDH1* mutant isogenic-U87 cell line has been widely used in glioma research (https://www.atcc.org/products/htb-14ig). Apart from U87, there is no other established glioma cell line as an isogenic pair differing just in the IDH1 status. Using unrelated cell lines (one IDH-WT and the other IDH-MUT) would rather obscure the results, as the observed differences could not be attributed to the IDH1 mutation, as the cells might accumulate other genetic alterations affecting transcriptional patterns. The findings in U87 were vastly verified using sequencing data from tumors. We performed detailed analyses of data collected in our lab (glioma Atlas [27]) and deposited in TCGA, providing more clinically relevant information.

Exploration of TCGA datasets showed increased *REST* expression in malignant gliomas and decreased *REST* expression in IDH-mutant when compared to IDH-WT gliomas (Fig. 1A, B). High *REST* expression has been reported as a negative prognostic factor for survival in GBM [9] and medulloblastoma patients [51]. In mice, expression of REST in glioma stem cells (GSCs) was negatively correlated with survival and considered as a critical factor in maintenance of their self-renewal [23]. While in all gliomas (LGG and GBM) *REST* expression was inversely correlated with patients survival as previously reported [23, 24, 52], we found that its high expression is an unfavorable prognostic factor in LGG with the *IDH* mutation, but a favorable factor in GBM (Fig. 1). Finding a positive correlation of *REST* expression with survival of GBM patients appears surprising and requires more studies.

Several studies reported that REST acts as an oncogene in gliomas, promoting cell proliferation and invasion [23, 24]. Some previous studies were done on 3D models [23] that were not performed in our study, however we aimed to relate our findings to observations from TCGA [29] or glioma Atlas [27]. REST expression was associated with high tumor aggressiveness and invasiveness, as well as chemotherapy resistance [23, 24, 52]. However, the underlying gene regulatory networks have not been elucidated yet. REST knockdown in U87 glioma cells affected many biological pathways. Numerous genes linked to neuronal functions were upregulated, while genes linked to cell proliferation were downregulated in REST depleted cells, regardless of the IDH status (Fig. 2E). However, a large subset of DEGs between IDH-WT and IDH-MUT U87 glioma cells were differently affected by REST knockdown (Fig. 3A). We found those genes belonged to GO biological processes related to ECM organization and glial/neuronal cell differentiation. Contrary to Zhang et al. observations [25], REST knockdown did not affect cell viability in our model, perhaps due to differences in experimental setup including transfection procedure, siRNA used, or type of proliferation assay, but it did influence cell invasiveness in IDH phenotype-specific manner. Zhang et al. observed decreased migration in the U87 cell line (IDH-WT), we observed the opposite (Fig. 4C). We also demonstrate a higher invasion of the IDH-MUT compared to IDH–WT cells (Fig. 4C), which is consistent with data on IDH-MUT tumors [53–55]. In our study, REST knockdown increased expression of ECM related genes (Fig. 4A) and invasion of IDH-MUT glioma cells (Fig. 4C), which coincided with changes in promoter and gene body methylation in more than half of the cases (Fig. 4D, E). These observations raised a question on how the changes in DNA methylation resulting from the *IDH* mutation affect REST targets and processes in which they are involved. We propose that REST-regulated genes from the ECM related biological pathway could contribute to the increased invasiveness of IDH-MUT gliomas.

The identified REST gene regulatory networks and biological functions agree with observations of a key role of REST as a repressor of neuronal genes in non-neuronal cells. REST depletion promotes neuronal differentiation [56], while REST stabilization promotes maintenance of NPCs [57]. However, ablation of REST expression to 1% of wild type levels appeared to impede the development of NSCs, NPCs and neurons [58]. REST regulates the timing of neural progenitor differentiation during neocortex development [59]. REST and CoREST modulate not only neuronal but also glial lineage [60]. Both REST and CoREST were found to target genes encoding factors involved in mediating glial cell identity and function [61]. Blocking the function of REST suppressed the nitric oxide-induced neuronal to glial switch in neural progenitor cells [62]. REST expression was upregulated and sustained by BMP signal activation in the course of astrocytic differentiation of NPCs, which restricted neuronal differentiation [63]. REST function is also required for the differentiation of OPCs into oligodendrocytes [64].

REST-depleted neural stem cells are defective in adherence, migration and survival [58]. Yet, REST involvement in cell migration and invasion can be seen as ambiguous. On one hand, REST blocks NPC radial migration during neurogenesis [59] and acts as a cell migration repressor in microglia [65]. However, medulloblastoma cells overexpressing REST migrated faster in wound-healing assay compared to controls [66]. One possible explanation of this seeming discrepancy is that cell migration in the brain can be tangential or radial [67] and REST may contribute differently to each of these types. In addition, the migratory pathway affected may reflect the differentiation status of the tumor.

Using DNA methylation data from U87 IDH-WT and IDH-MUT isogenic cell lines and tumor samples we determined how DNA hypermethylation affects REST regulated genes depending on IDH mutation status. REST ChIP-seq data on human U87 glioma cells integrated with the results of transcriptomic changes in REST depleted U87 cells allowed us to define direct and indirect REST targets. Scrutinizing REST ChIP-seq peaks we found REST binding motifs co-occurring with the binding sites for other TFs, among which KAISO was identified as an important partner in gene regulation. Interestingly, depending on the co-occurrence of REST and KAISO binding sites the effects on transcription varied and different GO biological pathways were found to be regulated. We confirmed that genes selected as REST targets in cultured glioma cells, were co-expressed in a REST dependent manner in TCGA glioma datasets.

The analysis of the REST-binding sites in ChIP-seq peaks shed light on different REST activity depending on the IDH status. The differences in the number of ChIP-seq peaks (Fig. 5A) and in genomic distribution of DNA binding sites (Fig. 5B) between IDH-WT and IDH-MUT suggest a stronger transcriptional regulation by REST (including both repression and activation) in IDH-WT glioma cells. Some REST ChIP-seq peaks were exclusive to IDH-WT or IDH-MUT cells, further adding up to the possible differences in REST regulation in IDH-WT and IDH-MUT. Moreover, the REST ChIP-seq peaks in IDH-WT and IDH-MUT cells contained differing sets of other transcription factor binding motifs (Additional File 5). Different occurrences of other TF motifs were also detected between peaks assigned to REST-repressed and -activated targets (Fig. 5 and Additional File 8) suggesting differences in gene regulation by specific factors [68–70]. Increased *REST* expression in G4 IDH-WT gliomas and decreased in IDH-MUT gliomas may result in REST binding to the sites that are otherwise occupied by other TFs with a different mode of action. Potential competitors of REST in regulation of its targets in IDH-WT and IDH-MUT cells included KAISO (ZBTB33), a methylation-sensitive TF [71–73].

The differential REST binding to DNA in IDH-WT and IDH-MUT cannot be explained by DNA methylation level within the REST ChIP-seq peaks specific to IDH-WT or IDH-MUT cells. The analyses in tumors show the expected increased DNA methylation in IDH-MUT samples (Additional File 6) but no specific DNA methylation pattern in loci corresponding to the REST peaks that would be related to their origin (specific to IDH-MUT, IDH-WT or common). Similarly, REST silencing did not significantly change DNA methylation pattern in these loci (Additional File 4). At the same time, DNA methylation within promoters or gene bodies is still an element that can significantly change the expression level of REST targets, as we have shown in the example of ECM related genes (Fig. 4). In general, this shows that the presence of REST does not have a stronger effect on the REST peaks methylation pattern than the IDH-related phenotype. Interestingly, the direction of change in the REST target genes expression seems to be decisive here, because we observed a significantly increased level of DNA methylation in REST motifs in the peaks assigned to repressed targets compared to activated targets (Fig. 8A-D).

KAISO is a transcriptional repressor reported in several different human cancers as a tumor suppressor or oncogene [74]. KAISO function seems to be highly context-dependent [74, 75]. It binds to methylated CpGs in two motifs containing a consensus sequence 5’-CGCG-3’ (shown in Fig. 5C, D: KAISO.0.A and KAISO.1.A) and to unmethylated C in the motifs with another consensus sequence 5’-CTGCNA-3’ (Fig. 5D: KAISO.2.A) [75]. We found that KAISO TF motifs discriminate between the REST binding sites specific for IDH-WT and IDH-MUT cells. All three KAISO motifs were found in a number of REST ChIP-seq peaks specific for IDH-WT and common for IDH-MUT and IDH-WT cells. Contrary, not a single KAISO motif was detected among the motifs present in the REST ChIP-seq peaks specific for IDH-MUT cells (Additional File 5). Depending on the co-occurrence of REST and KAISO binding sites, the effects on transcription and DNA methylation patterns varied (Figs. 8A-E, 6E) and different GO biological pathways were found to be regulated (Additional File 8A, B). In addition, the KAISO-binding motif 2.A, which is bound by KAISO when unmethylated, was among the top motifs found in REST-activated but not in REST-repressed genes (Fig. 6B, 6E).

While the repressive role of REST has been well documented, its role in gene activation is less described. As KAISO appeared in our TF motif analysis as a putative co-regulator of REST-activated genes, we performed KAISO silencing experiments as well. We found that genes repressed by REST are more easily perturbed with REST knockdown, but strikingly, more REST-activated genes were perturbed with KAISO silencing (Fig. 7C, D). Additionally, there was a considerable overlap between genes upregulated by REST and KAISO silencing in both IDH-MUT and IDH-WT (Fig. 7E, G), which supports the hypothesis on the synergistic repressive effect of REST and KAISO on expression of these genes. Situation was different for REST-activated genes, where silencing of REST or KAISO caused opposite effects on gene expression for a high proportion of genes (Fig. 7F, H), suggesting competition between these TFs. Lastly, the top GO BP pathway that was upregulated by siKAISO within REST-activated genes was ECM organization (the genes included in this pathway are highlighted in Fig. 7J), a pathway that was significantly affected by REST silencing as well.

Regulation of gene expression by REST is complex and includes a variety of cofactors, including mammalian SIN3 transcription regulator family member A (mSin3a) and REST corepressor 1 (CoREST). This complex mediates the recruitment of additional chromatin modulators, such as histone deacetylases (HDAC1/2), histone demethylases (G9a, SUV39H1, LSD1), MeCP2, and the DNA methyltransferase DNMT1, and generally leads to repression of gene expression [16, 73]. Since KAISO itself enables the recruitment of the DNA methylation modifying machinery [76, 77] it is possible that when REST and KAISO occupy overlapping sites on DNA, they may compete for binding to DNA, or may interfere with each other’s influence on DNA methylation.

Intersection of the genes with assigned REST ChIP-seq peaks with those affected by REST knockdown uncovered a number of genes that are high confidence primary targets of REST (Fig. 5). Among genes upregulated in REST depleted cells we found genes related to signal release, calcium regulated exocytosis and telencephalon glial cell migration as identified with the REACTOME enrichment analysis. Most of these genes showed decreased expression in G2 and G3 gliomas compared to normal brain samples, with the lowest expression in G4 gliomas (Additional File 7C). The expression of these genes was negatively correlated with signatures of G1S and G2M phases of the cell cycle, and positively correlated with NPC-like cellular states as defined by Neftel et al. [26]. NPC-like cellular states are enriched in the proneural GBM [26], originally defined as IDH-MUT GBMs or secondary GBMs (in WHO 2021 classification they would be called G4 astrocytomas) [78]. Our findings suggest that REST plays a role specifically in these malignant gliomas. Intermediate expression of *REST* in G2/G3 gliomas (Fig. 1A) may be enough to maintain a higher expression of the genes contributing to the NPC-like state. High *REST* expression in G4 gliomas might be associated with a strong repression of these genes. This could explain the seemingly opposite effects of REST: REST is a negative prognostic factor in G2/G3 gliomas, and a positive factor in G4 gliomas.

## Conclusions

In summary, we identified REST targets, gene regulatory networks and putative REST cooperativity with other TFs that differentially control gene expression in IDH-WT and IDH-MUT gliomas. Among REST targets we found genes involved in glial cell differentiation and ECM organization. Knockdown of REST had a different impact on glioma invasion depending on the IDH phenotype, which is connected to DNA hypermethylation phenotype. We demonstrate that activation or repression of REST-mediated gene transcription might be differentially modulated by DNA methylation and by cooperation/competition with other transcription factors, such as KAISO (ZBTB33). The DNA methylation of REST activated genes often showed a positive correlation with gene expression, suggesting that the hypermethylation phenotype of IDH-MUT may have a strong impact on these genes. Finally, repression of the canonical REST gene targets may play a more significant role in IDH-MUT grade 2/3 gliomas than in G4 gliomas by maintaining NPC-like cellular state properties. Therefore, REST could be considered as a potential factor in the design of targeted glioma therapies.

### Supplementary Information

**Additional file 1.** Differences in gene expression between U87 IDH-MUT and IDH-WT. **A** Volcano plot U87 IDH-MUT vs IDH-WT gene expression comparison. Genes on the right-hand side of the plot have higher expression in IDH-MUT compared to IDH-WT, while genes on the left-hand of the plot have higher expression in IDH-WT compared to IDH-MUT. **B** REACTOME pathways analysis of genes expressed differentially between U87 IDH-MUT and IDH-WT glioma cell lines. **C** Comparison of the REACTOME pathway enrichment for the genes upregulated in IDH-MUT glioma in U87 (IDH-MUT vs. IDH-WT, left column) and TCGA (G2/G3 IDH-MUT versus IDH-WT gliomas, right column). **D** Comparison of the REACTOME pathway enrichment for the genes downregulated in IDH-MUT glioma in U87 (IDH-MUT vs. IDH-WT, left column) and TCGA (IDH-MUT G2/G3 versus IDH-WT gliomas, right column). **E** Kaplan-Meier overall survival curves for the patients with WHO grade 4 glioma IDH-WT (n=55 high REST; n=55 low REST) and **F** G4 astrocytomas IDH-MUT (n=14 high REST; n=14 low REST). The patients were stratified into groups based on high or low REST expression levels. For patients still alive at the time of analysis, their data were censored at the time of the last follow-up. Statistical significance was assessed using the Log Rank Test.

**Additional file 2.** Comparison of IDH-MUT in U87 cell lines and primary human G4 gliomas. **A** Sorted values of log2 fold change (log2 FC) for the genes coming from the IDH-MUT vs IDH-WT comparison in U87 cell lines were presented as red dots. Values for the same genes coming from IDH-MUT vs IDH-WT comparison in primary cell lines were overlaid as gray dots. Percent of log2 FC direction concordance between IDH-MUT vs IDH-WT in U87 cell lines and primary cell lines was calculated. Number of differentially expressed genes is indicated. Black vertical line separates genes expressed higher in IDH-MUT U87 compared to IDH-WT U87 from the genes expressed higher in IDH-WT U87 compared to IDH-MUT U87. **B **Genes assigned to extracellular matrix (ECM) organization in Fig. 3 and downregulated in U87 IDH-MUT vs IDH-WT were presented as z-score heatmap. **C** The same genes were visualized using in-house primary IDH-MUT/WT cell lines. In case, when gene was significantly downregulated an asterisk was appended to its name (*p.adjusted<0.05, **p. adjusted<0.01, ***p.adjusted<0.001). **D** Bootstrapping result, where significance of obtaining by chance 13 out of 18 significantly downregulated genes was evaluated.

**Additional file 3.** Heatmap of the transcript levels of the genes related to extracellular matrix organization GO biological pathway shown in Fig. 4C (genes expressed differentially in IDH WT and MUT glioma and modulated by siREST). The columns (patients) were sorted according to decreasing REST gene expression in the tumor sample; left hand side heatmap shows GBM dataset, middle shows LGG IDH-MUT and right-hand side heatmaps shows LGG IDH-WT samples. Asterisk (*) mark genes that had significant correlation of expression with *REST* gene expression in TCGA glioma dataset.

**Additional file 4.** REST ChIP-seq peaks DNA methylation level, of U87 IDH-WT and U87 IDH-MUT cell lines samples treated with siCTRL and siREST, in a resolution of single cytosine loci. List of REST repressed or REST activated genes containing differentially methylated sites in REST or KAISO motifs within the associated REST ChiPseq peaks. **A** Difference versus log10 fold change (FC) in DNA methylation between siREST and siCTRL samples of U87 IDH-WT cell line; **B** as in A but for U87 IDH-MUT cell line; **C** Difference versus FC in DNA methylation between siCTRL IDH-MUT and siCTRL WT samples; **D** as in C but for siREST IDH-MUT and siREST IDH-WT samples; In **A**-**D**: light gray - no difference, red - decreased methylation (difference ≤ − 0.15), green - increased methylation (difference ≥ 0.15); **E** Difference in cytosine methylation between U87 IDH-MUT and U87 IDH-WT cell lines in siREST and siCTRL samples; **F** Cytosines within REST ChIP-seq peaks were divided into three categories (WT-specific, MUT-specific, common) and presented as percentage of single cytosines that overlapped with one of the three types of REST ChIP-seq peaks. “Background” refers to all cytosines within REST-peaks, “Differential” represents non-light gray cytosines shown **C**&**D**, “Higher” (red) - cytosines with higher DNA methylation in IDH-MUT, "Lower" (green) - cytosines with lower DNA methylation in IDH-MUT; **G** log10 fold change between percentage of “Differential” cytosines to “Background” cytosines in each of the three REST ChIP-seq peaks categories: common, WT-, MUT-specific.

**Additional file 5.** Analysis of genes assigned to REST ChIP-seq peaks specific for IDH-MUT or IDH-WT U87 cells. **A** Differential REACTOME analysis of genes annotated uniquely to either IDH-MUT or IDH-WT REST ChIP-seq peaks. U87 IDH-MUT or IDH-WT promoters (+/-3kB from transcription start site - TSS) peaks were an input to ChIPseeker analysis with compareCluster function. **B** Venn diagram showing the number of TF motifs found in the REST ChIP-seq peaks located within gene promoters in only IDH-WT (red), only IDH-MUT (purple) or both in IDH-WT and IDH-MUT (yellow). **C **EnrichR results presenting which TFs based on the ENCODE data were found at genomic positions where REST ChIP-seq peaks were identified in U87 IDH-MUT and U87 IDH-WT. The top panel (yellow) presents results for the peaks common to both IDH-WT and IDH-MUT, the middle (red) - for IDH-WT specific REST ChIP-seq peaks, and the bottom one (purple) for IDH-MUT specific REST ChIP-seq peaks. **D** REST and KAISO (ZBTB33) motif occurrence within the gene promoters bound in REST ChIP-seq. Venn diagrams show the number of gene promoter sequences that contained REST or KAISO motifs and were (I) common between IDH-WT and IDH-MUT, (II) unique to IDH-WT, and (III) unique to IDH-MUT.

**Additional file 6.** DNA methylation pattern of the REST ChIP-seq peaks (summit ± 100bp) in tumors (glioma Atlas). REST ChIP-seq peaks **A** common, **B** IDH-MUT-specific or **C** IDH-WT-specific that were differentially methylated in the following pairs of glioma tumor types; **D-E** Mean REST ChIP-seq peak DNA methylation of the peaks differentially methylated between G2/G3 IDH-WT and IDH-MUT glioma samples. Heatmaps are arranged in order of decreasing difference in methylation between G2/G3 IDH-WT and IDH-MUT glioma samples; **D** Peaks common for IDH-WT and IDH-MUT (n=128); **E** REST ChIP-seq peaks unique to IDH-MUT (n=4); **F **REST ChIP-seq peaks unique to IDH-WT (n=32).

**Additional file 7.** GO Biological Pathway enrichment for the genes identified in REST ChIP-seq and and REST knockdown experiments. **A **GO BP analysis for the genes that were upregulated or **B** downregulated by siREST and targeted by REST in the ChIP-seq experiment. ChIP-seq experiments on both U87 IDH-MUT, IDH-WT cell lines and from freshly resected human tumors show a good overlap of the number of genes annotated to ChIP-seq peaks. **C** Expression of genes upregulated in REST depleted cells (from panel A) in TCGA LGG/GBM datasets and presented as bee swarm plots for NB (normal brain), glioma WHO G2, G3 and G4.

**Additional file 8.** Characterization of ChIP-seq peaks and TF motifs in REST-repressed and REST-activated genes. **A **Gene Ontology Biological Process gene enrichment analysis for repressed REST targets (left panel) and activated REST targets (right panel). **B **Clustering of TF motifs characteristic for genes activated by REST, repressed by REST, and common to both REST-activated and REST-repressed, based on TF PWMs. Distribution of KAISO motifs within the promoters of the genes activated (**C**) and repressed (**D**) by REST. **E **Distribution of REST motifs within the promoters of the genes repressed by REST. **F** DNA sequence logos of the REST binding motifs PWMs; **G** DNA sequence logos of the KAISO binding motifs PWMs.

**Additional file 9.** ZBTB33 (KAISO) silencing experiment. **A **Relative expression of** ZBTB33 **(coding KAISO) in IDH-WT and IDH-MUT U87 cells at 72 hours of silencing with siRNA. mRNA levels in transfected cells were determined by quantitative PCR and normalized to *GAPDH* expression in the same sample, and expressed as the fold change relative to the siCTRL. Data are represented as mean ± SD, n = 4 independent experiments, **p<0.01, ***p<0.001, one way ANOVA. **B** Representative immunoblots illustrating levels of KAISO and reference proteins (mutated IDH1, REST, Histone H3, GAPDH and beta-actin) in IDH-WT and IDH-MUT U87 cells at 72 hours after knockdown of KAISO determined by Western blotting. **C **Relative expression of** MMP7** in IDH-WT and IDH-MUT U87 cells at 72 hours of *ZBTB33* (coding KAISO) silencing with siRNA. mRNA levels in transfected cells were determined by quantitative PCR and normalized to *GAPDH* expression in the same sample and expressed as the fold change relative to the siCTRL. Data are represented as mean ± SD, n = 4 independent experiments, *p<0.05, one way ANOVA.

**Additional file 10.** REST and KAISO silencing additional information. **A** Bootstrapping results for siKAISO experiment, where number of siKAISO DEGs was intersected with REST-repressed and REST-activated gene categories separately in IDH-MUT and IDH-WT samples (red dots) and compared to random sampling experiment were the same number of genes as number of genes in REST-activated or REST-repressed was drawn 1000 times and checked how many of them appeared as DEGs in our siKAISO experiment (gray dots). **B** The same bootstrapping experiment as described above but for siREST experiment. **C** Venn diagrams of genes up- or downregulated by siREST treatment in IDH-WT or IDH-MUT cells. **D** Venn diagrams of genes up- or downregulated by siKAISO treatment in IDH-WT or IDH-MUT cells. **E** Plots of 6 last deciles (out of 10 total) of log2 of variance from normalized counts for siCTRL for IDH-MUT and IDH-WT and siREST IDH-MUT and IDH-WT. **F** Plots of 6 last deciles (out of 10 total) of log2 of variance from normalized counts for siCTRL for IDH-MUT and IDH-WT and siKAISO IDH-MUT and IDH-WT.

**Additional file 11.** Results of chi2 test and post hoc test performed on the data presented on Fig. 7CD.

**Additional file 12.** Table presenting Fisher’s exact test analysis using Gene Ontology Biological Processed of genes up-regulated by siKAISO and intersecting with REST-activated genes (related to Fig. 7J).

**Additional file 13.** Number of cytosines within REST or KAISO motifs within REST ChIP-seq peaks assigned to REST-repressed or -activated targets. The peaks were categorized according to whether they had detected binding motifs of both REST and KAISO, or just one of them.

**Additional file 14.** Number of sites where individual REST or KAISO motifs were detected within REST ChIP-seq peaks derived from the cell lines. Sites discovered as differentially methylated among glioma tumor groups were matched to individual REST or KAISO motifs and then to peaks that were paired with REST-repressed or -activated targets. The number of all detected sites of REST or KAISO motifs and the number of differentially methylated sites were significantly different (Chi2 = 34.763, df = 14, *p*-value = 0.001593). Asterisk indicates differences in DNA methylation larger than expected by chance (*). ND - no data.

**Additional file 15.** List of REST repressed or REST activated genes containing differentially methylated sites in REST or KAISO motifs within the associated REST ChiP-seq peaks.

## Data Availability

The datasets generated and/or analyzed during the current study are available in the NCBI Gene Expression Omnibus repository (https://www.ncbi.nlm.nih.gov/geo/query/acc.cgi?acc=GSE174308, REST transcription factor holds the balance between the invasion and cell differentiation in IDH-mutant and IDH-wild type gliomas) and in European Genome-Phenome Archive (EGAD00001008986—Glioma specimens of both IDH-MUT and IDH-WT REST ChIPseq data).

## Abbreviations

5mC: 5-Methylcytosine
α-KG: α-Ketoglutarate
AC-like: Astrocyte-like
BMP: Bone morphogenetic protein
ChIP-seq: Chromatin immunoprecipitation-sequencing
coREST: Corepressor REST
CpG: Cytosine phosphate guanine
dDEG: Decreased DEG (a gene differentially expressed in IDH-WT and IDH-MUT that had a lower fold change in siREST-transfected cells than in siCTRL transfected cells)
DEG: Differentially expressed gene
ECM: Extracellular matrix
ENCODE: The Encyclopedia of DNA Elements
GBM: Glioblastoma, WHO grade IV
G2: WHO grade 2
G3: WHO grade 3
G4: WHO grade 4
GO: Gene Ontology
GSC: Glioma stem cell
HOCOMOCO: HOmo sapiens COmprehensive MOdel COllection
iDEG: Increased DEG (genes differentially expressed in IDH-WT and IDH-MUT that had a higher fold change in siREST-transfected cells than in siCTRL transfected cells)
IDH (1/2): Isocitrate dehydrogenase (1 or 2)
IDH-MUT: Mutated isocitrate dehydrogenase 1 or 2
IDH-WT: IDH wild type samples (neither IDH1 nor IDH2 were mutated)
LGG: Lower grade gliomas, WHO grades 2 and 3
MES-like: Mesenchymal-like
NB: Normal brain
NPC: Neural progenitor cell
NSC: Neuronal stem cells
OPC: Oligodendrocyte-progenitor cell
PNET: Primitive neuroepithelial tumors
PWM: Position weighted matrix
RE1: Repressor element-1
REST: Repressor element-1-silencing transcription factor / RE1 silencing transcription factor/neural-restrictive silencing factor (also known as neuron-restrictive silencer factor NRSF)
siRNA: Short interfering RNA
TCGA: The Cancer Genome Atlas
TF: Transcription factor
ZBTB33: Zinc Finger and BTB Domain Containing 33 transcription factor (also known as KAISO)
