# Supplementary figures and images for: Comprehensive analysis of the REST transcription factor regulatory networks in IDH mutant and IDH wild-type glioma cell lines and tumors
Source: Acta Neuropathol Commun. 2024 May 6;12:72. doi: 10.1186/s40478-024-01779-y (PMC11071216; doi:10.1186/s40478-024-01779-y)

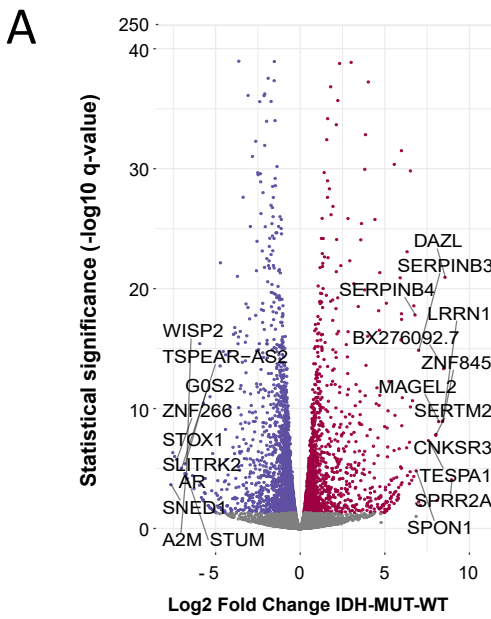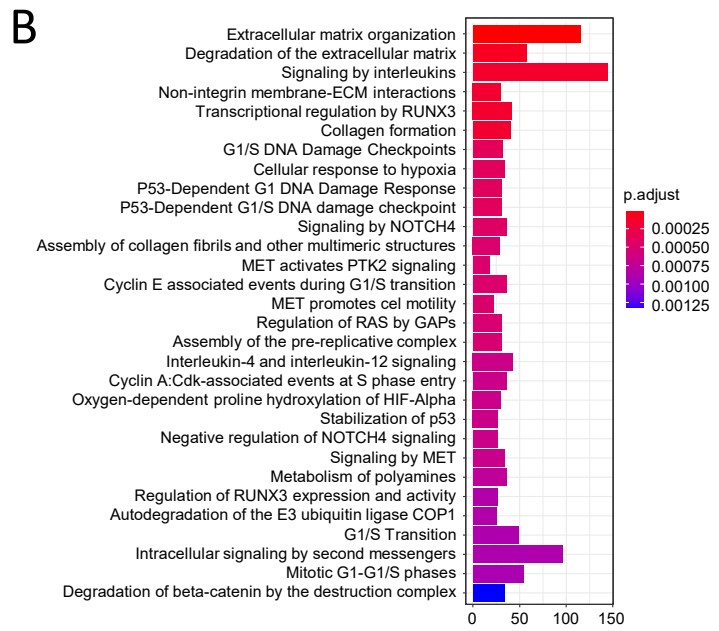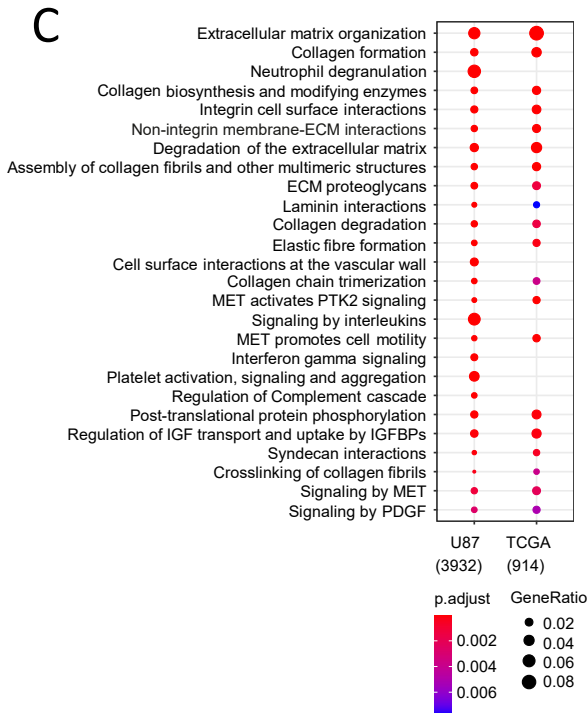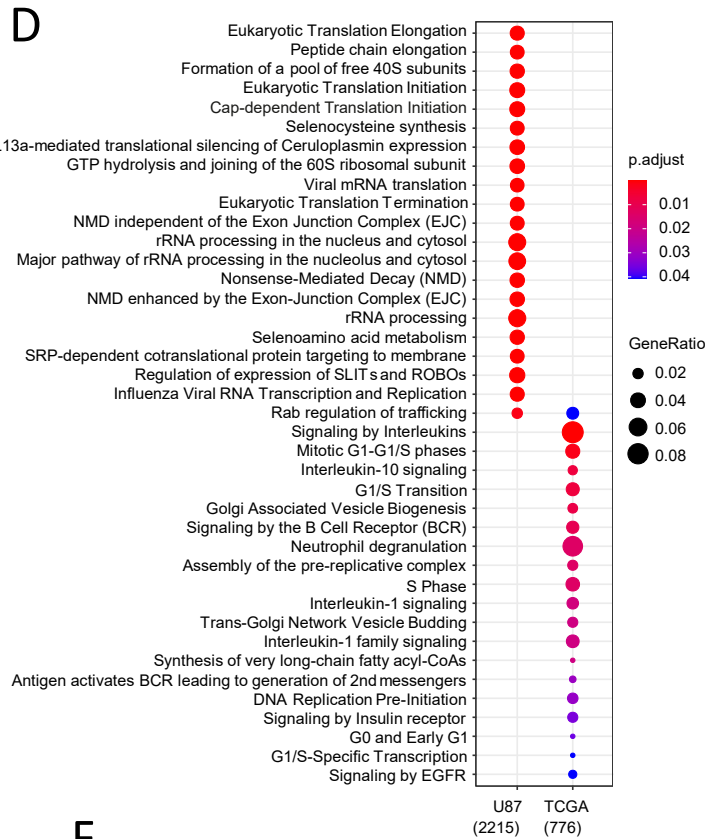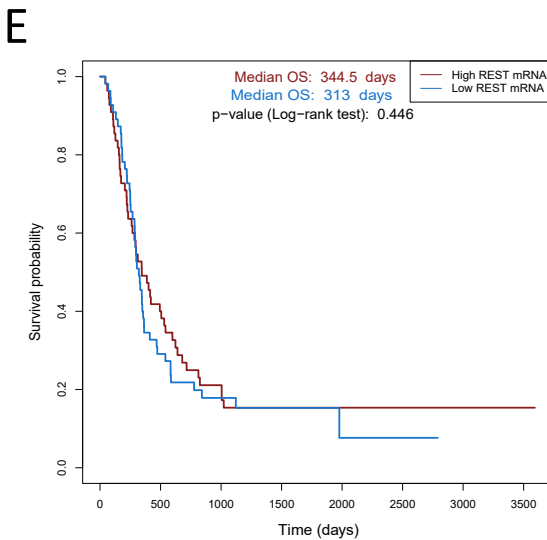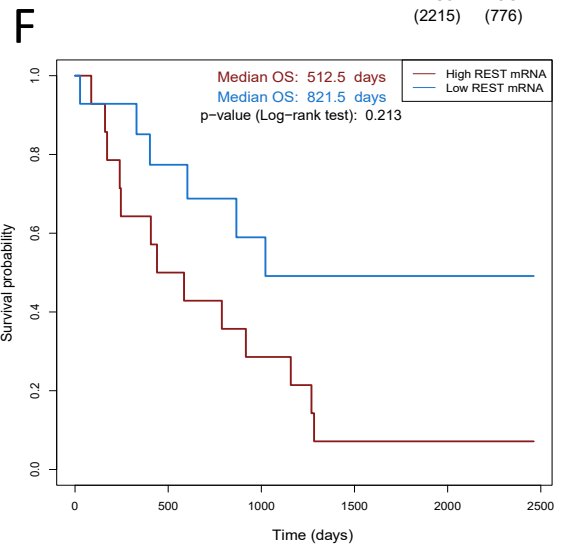

Supplement: Supplementary file 1 — Additional file 1. Differences in gene expression between U87 IDH-MUT and IDH-WT. A Volcano plot U87 IDH-MUT vs IDH-WT gene expression comparison. Genes on the right-hand side of the plot have higher expression in IDH-MUT compared to IDH-WT, while genes on the left-hand of the plot have higher expression in IDH-WT compared to IDH-MUT. B REACTOME pathways analysis of genes expressed differentially between U87 IDH-MUT and IDH-WT glioma cell lines. C Comparison of the REACTOME pathway enrichment for the genes upregulated in IDH-MUT glioma in U87 (IDH-MUT vs. IDH-WT, left column) and TCGA (G2/G3 IDH-MUT versus IDH-WT gliomas, right column). D Comparison of the REACTOME pathway enrichment for the genes downregulated in IDH-MUT glioma in U87 (IDH-MUT vs. IDH-WT, left column) and TCGA (IDH-MUT G2/G3 versus IDH-WT gliomas, right column). E Kaplan-Meier overall survival curves for the patients with WHO grade 4 glioma IDH-WT (n=55 high REST; n=55 low REST) and F G4 astrocytomas IDH-MUT (n=14 high REST; n=14 low REST). The patients were stratified into groups based on high or low REST expression levels. For patients still alive at the time of analysis, their data were censored at the time of the last follow-up. Statistical significance was assessed using the Log Rank Test. [file 40478_2024_1779_MOESM1_ESM.pdf]

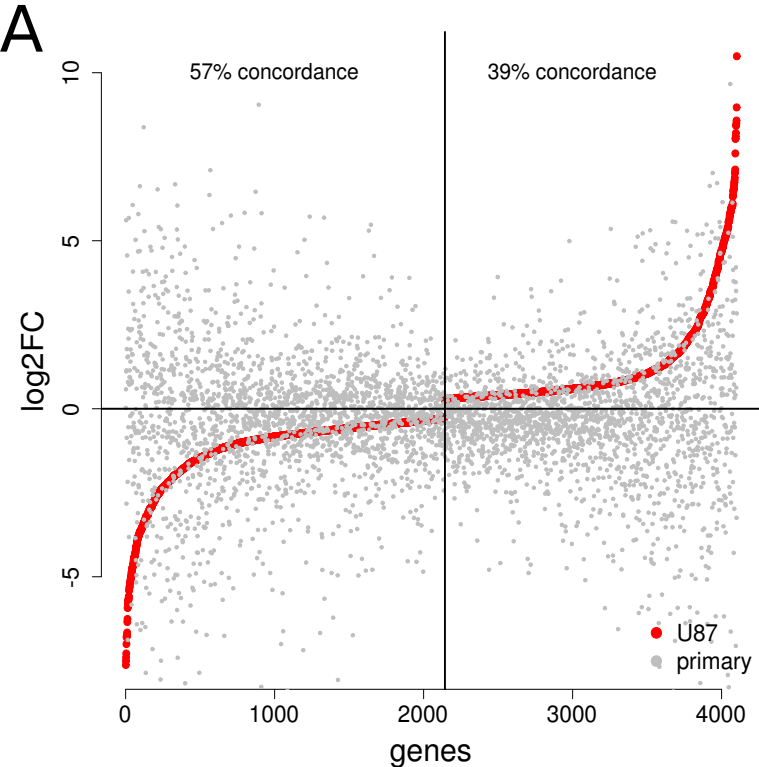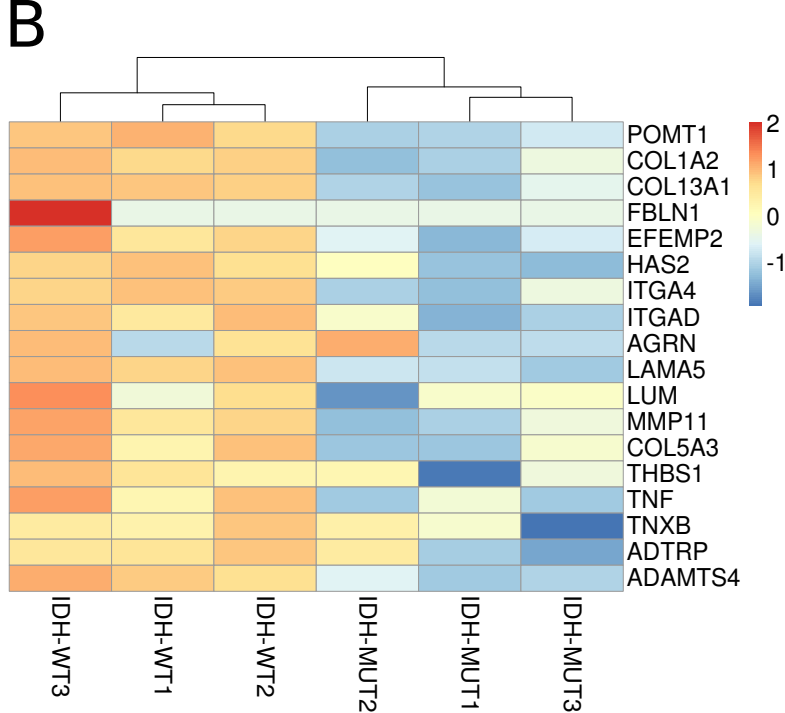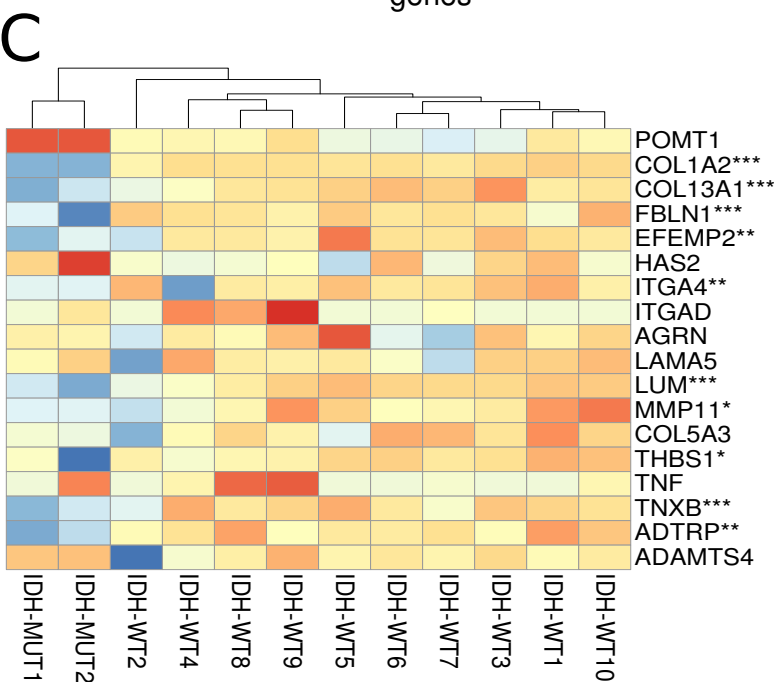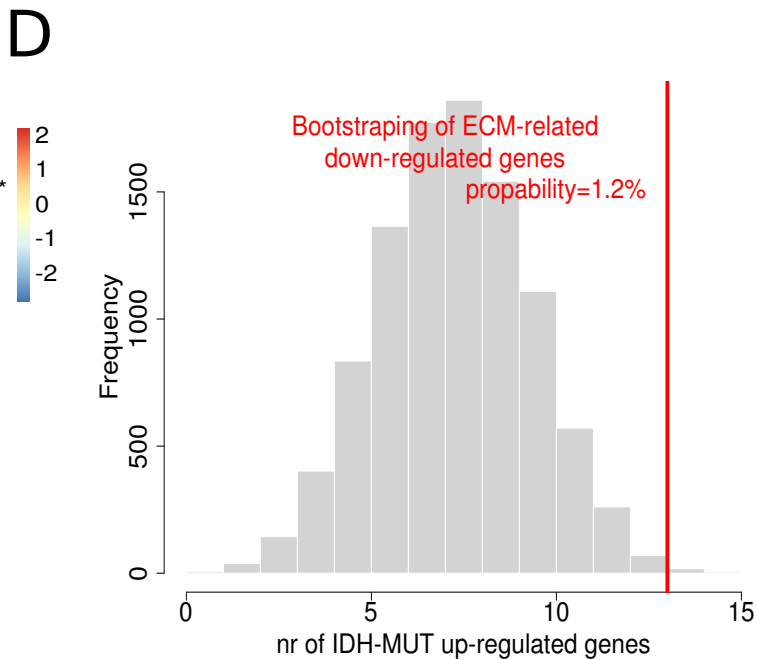

Supplement: Supplementary file 2 — Additional file 2. Comparison of IDH-MUT in U87 cell lines and primary human G4 gliomas. A Sorted values of log2 fold change (log2 FC) for the genes coming from the IDH-MUT vs IDH-WT comparison in U87 cell lines were presented as red dots. Values for the same genes coming from IDH-MUT vs IDH-WT comparison in primary cell lines were overlaid as gray dots. Percent of log2 FC direction concordance between IDH-MUT vs IDH-WT in U87 cell lines and primary cell lines was calculated. Number of differentially expressed genes is indicated. Black vertical line separates genes expressed higher in IDH-MUT U87 compared to IDH-WT U87 from the genes expressed higher in IDH-WT U87 compared to IDH-MUT U87. B Genes assigned to extracellular matrix (ECM) organization in Fig. 3 and downregulated in U87 IDH-MUT vs IDH-WT were presented as z-score heatmap. C The same genes were visualized using in-house primary IDH-MUT/WT cell lines. In case, when gene was significantly downregulated an asterisk was appended to its name (*p.adjusted<0.05, **p. adjusted<0.01, ***p.adjusted<0.001). D Bootstrapping result, where significance of obtaining by chance 13 out of 18 significantly downregulated genes was evaluated. [file 40478_2024_1779_MOESM2_ESM.pdf]

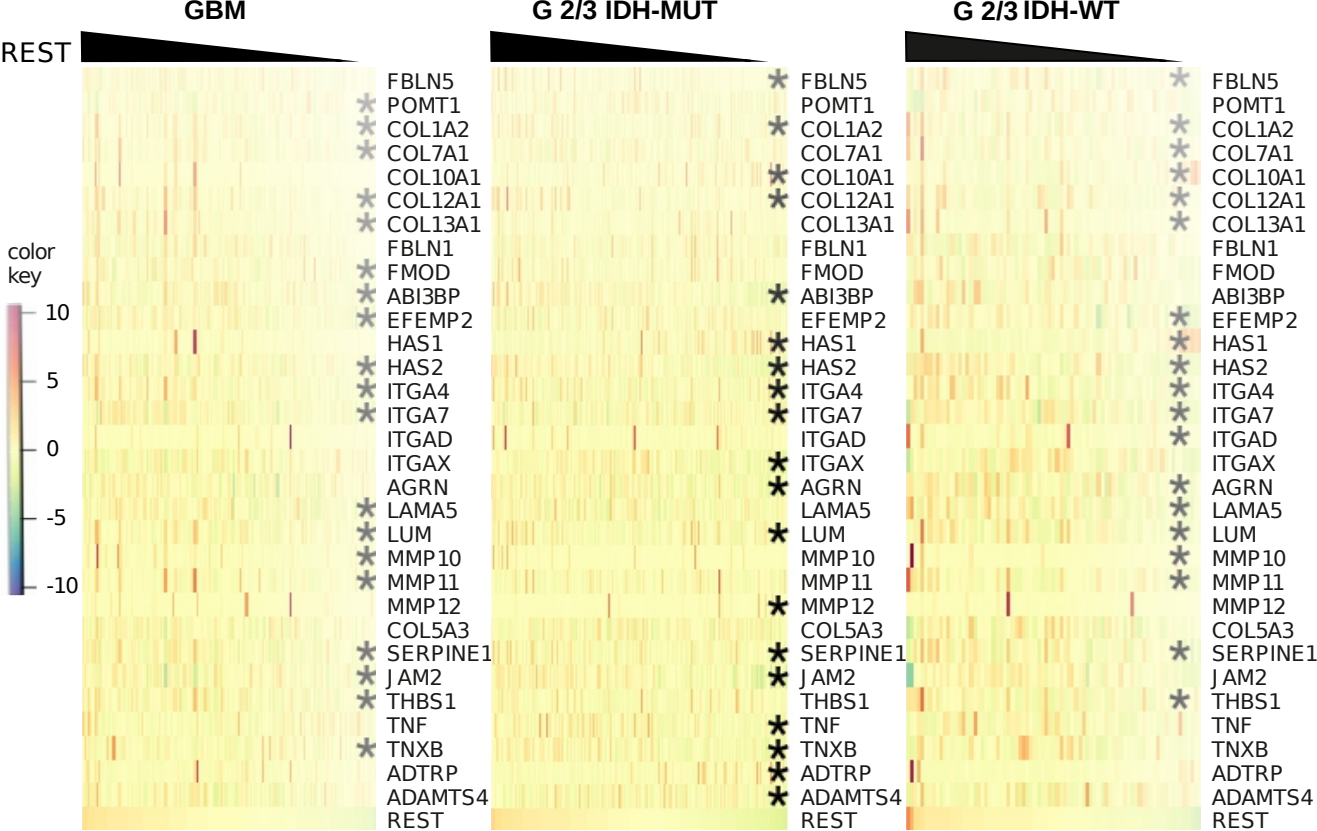

Supplement: Supplementary file 3 — Additional file 3. Heatmap of the transcript levels of the genes related to extracellular matrix organization GO biological pathway shown in Fig. 4C (genes expressed differentially in IDH WT and MUT glioma and modulated by siREST). The columns (patients) were sorted according to decreasing REST gene expression in the tumor sample; left hand side heatmap shows GBM dataset, middle shows LGG IDH-MUT and right-hand side heatmaps shows LGG IDH-WT samples. Asterisk (*) mark genes that had significant correlation of expression with REST gene expression in TCGA glioma dataset. [file 40478_2024_1779_MOESM3_ESM.pdf]

A

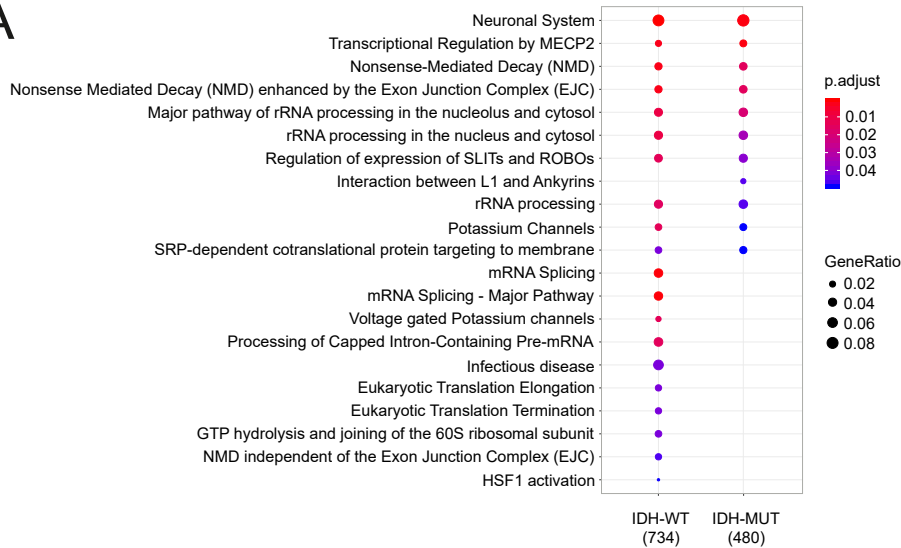

B

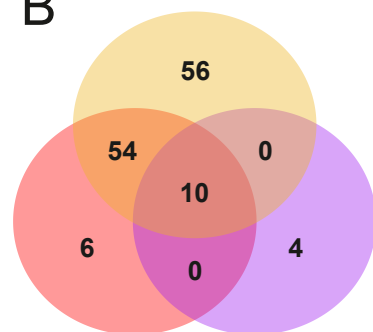

C

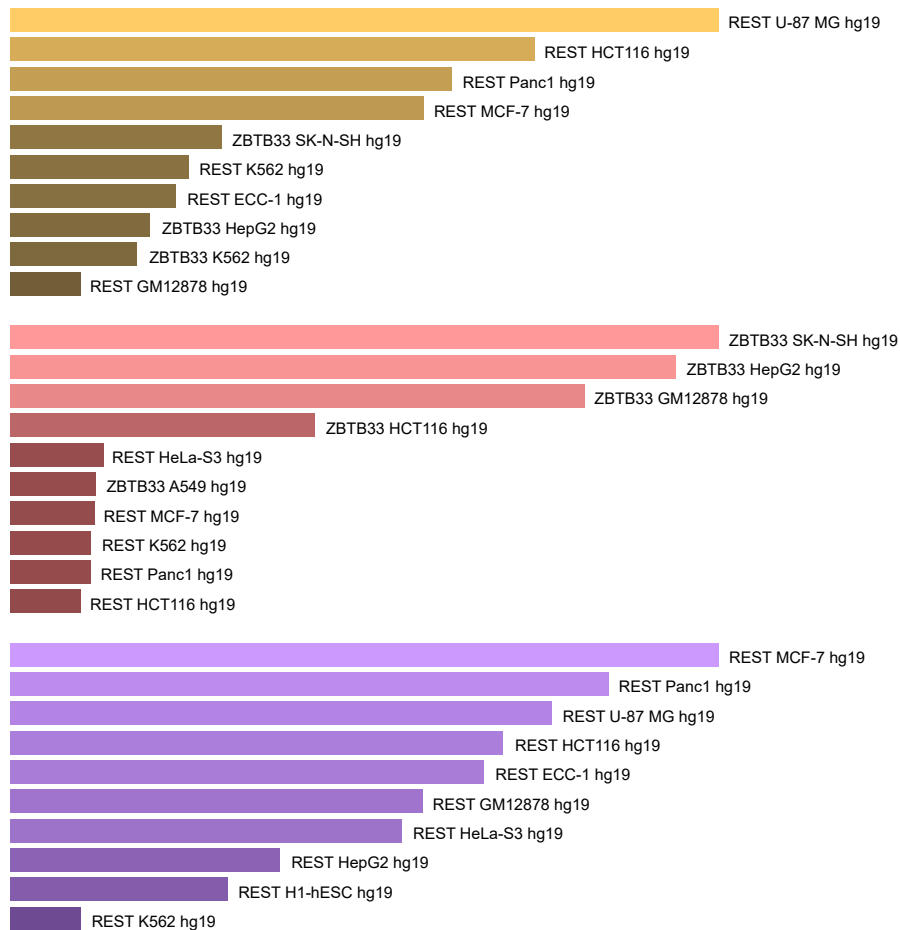

D

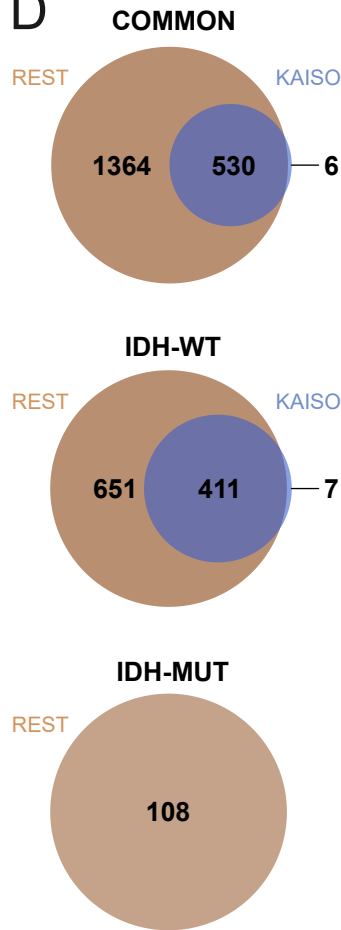

Supplement: Supplementary file 5 — Additional file 5. Analysis of genes assigned to REST ChIP-seq peaks specific for IDH-MUT or IDH-WT U87 cells. A Differential REACTOME analysis of genes annotated uniquely to either IDH-MUT or IDH-WT REST ChIP-seq peaks. U87 IDH-MUT or IDH-WT promoters (+/-3kB from transcription start site - TSS) peaks were an input to ChIPseeker analysis with compareCluster function. B Venn diagram showing the number of TF motifs found in the REST ChIP-seq peaks located within gene promoters in only IDH-WT (red), only IDH-MUT (purple) or both in IDH-WT and IDH-MUT (yellow). C EnrichR results presenting which TFs based on the ENCODE data were found at genomic positions where REST ChIP-seq peaks were identified in U87 IDH-MUT and U87 IDH-WT. The top panel (yellow) presents results for the peaks common to both IDH-WT and IDH-MUT, the middle (red) - for IDH-WT specific REST ChIP-seq peaks, and the bottom one (purple) for IDH-MUT specific REST ChIP-seq peaks. D REST and KAISO (ZBTB33) motif occurrence within the gene promoters bound in REST ChIP-seq. Venn diagrams show the number of gene promoter sequences that contained REST or KAISO motifs and were (I) common between IDH-WT and IDH-MUT, (II) unique to IDH-WT, and (III) unique to IDH-MUT. [file 40478_2024_1779_MOESM5_ESM.pdf]

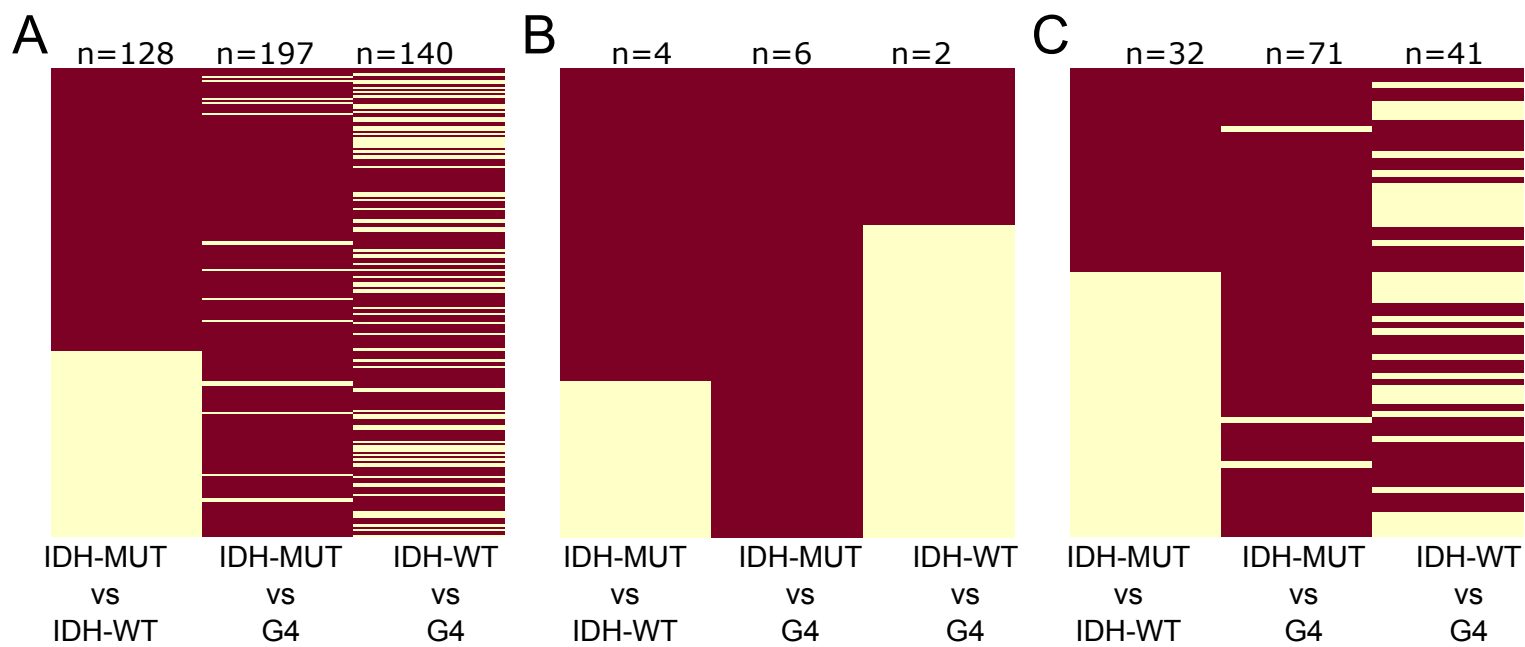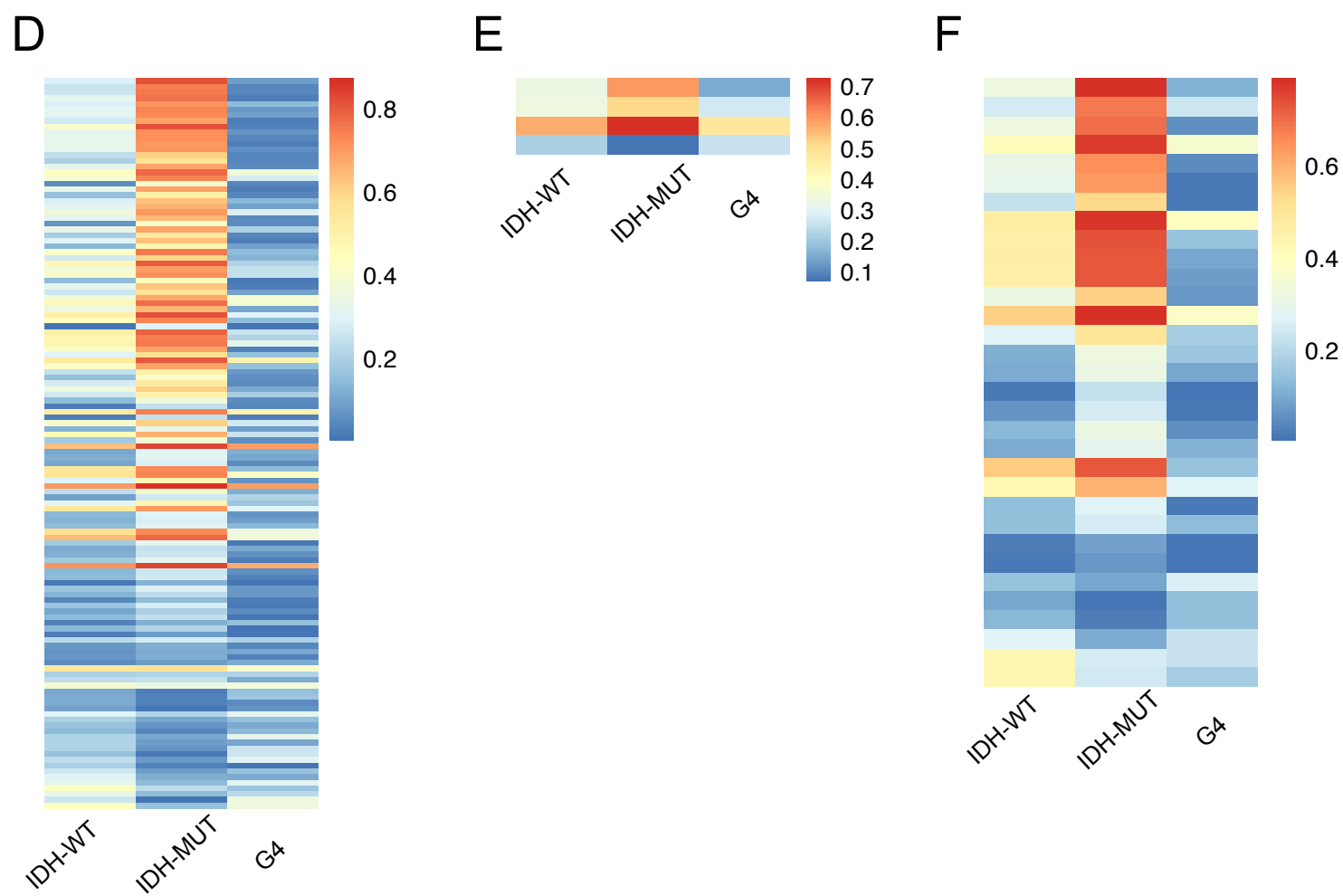

Supplement: Supplementary file 6 — Additional file 6. DNA methylation pattern of the REST ChIP-seq peaks (summit ± 100bp) in tumors (glioma Atlas). REST ChIP-seq peaks A common, B IDH-MUT-specific or C IDH-WT-specific that were differentially methylated in the following pairs of glioma tumor types; D-E Mean REST ChIP-seq peak DNA methylation of the peaks differentially methylated between G2/G3 IDH-WT and IDH-MUT glioma samples. Heatmaps are arranged in order of decreasing difference in methylation between G2/G3 IDH-WT and IDH-MUT glioma samples; D Peaks common for IDH-WT and IDH-MUT (n=128); E REST ChIP-seq peaks unique to IDH-MUT (n=4); F REST ChIP-seq peaks unique to IDH-WT (n=32). [file 40478_2024_1779_MOESM6_ESM.pdf]

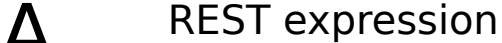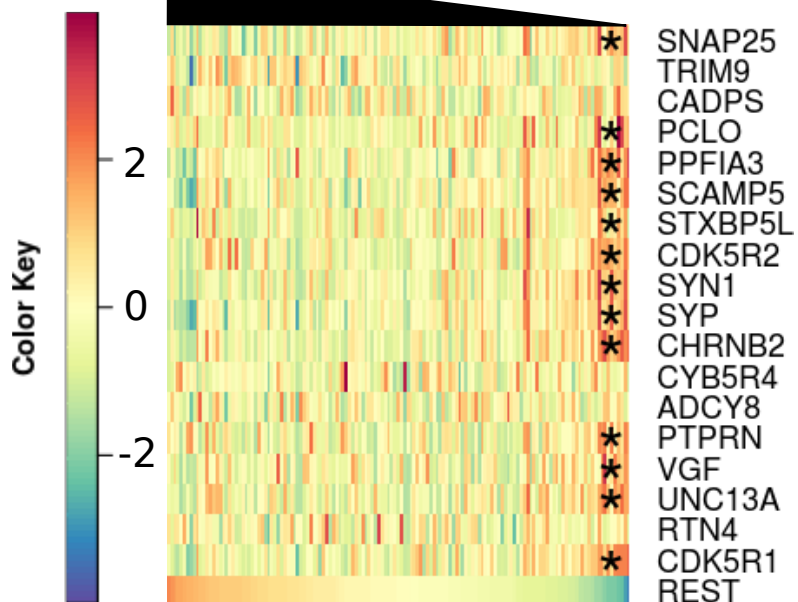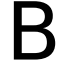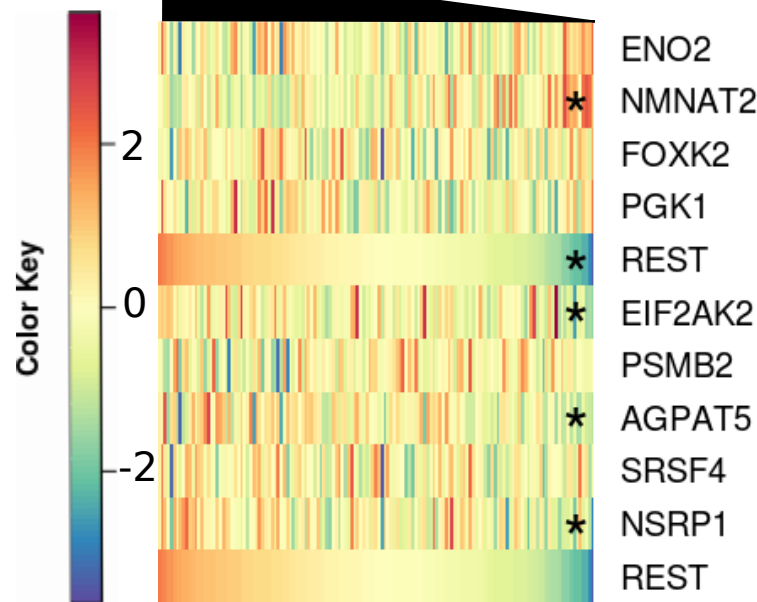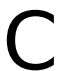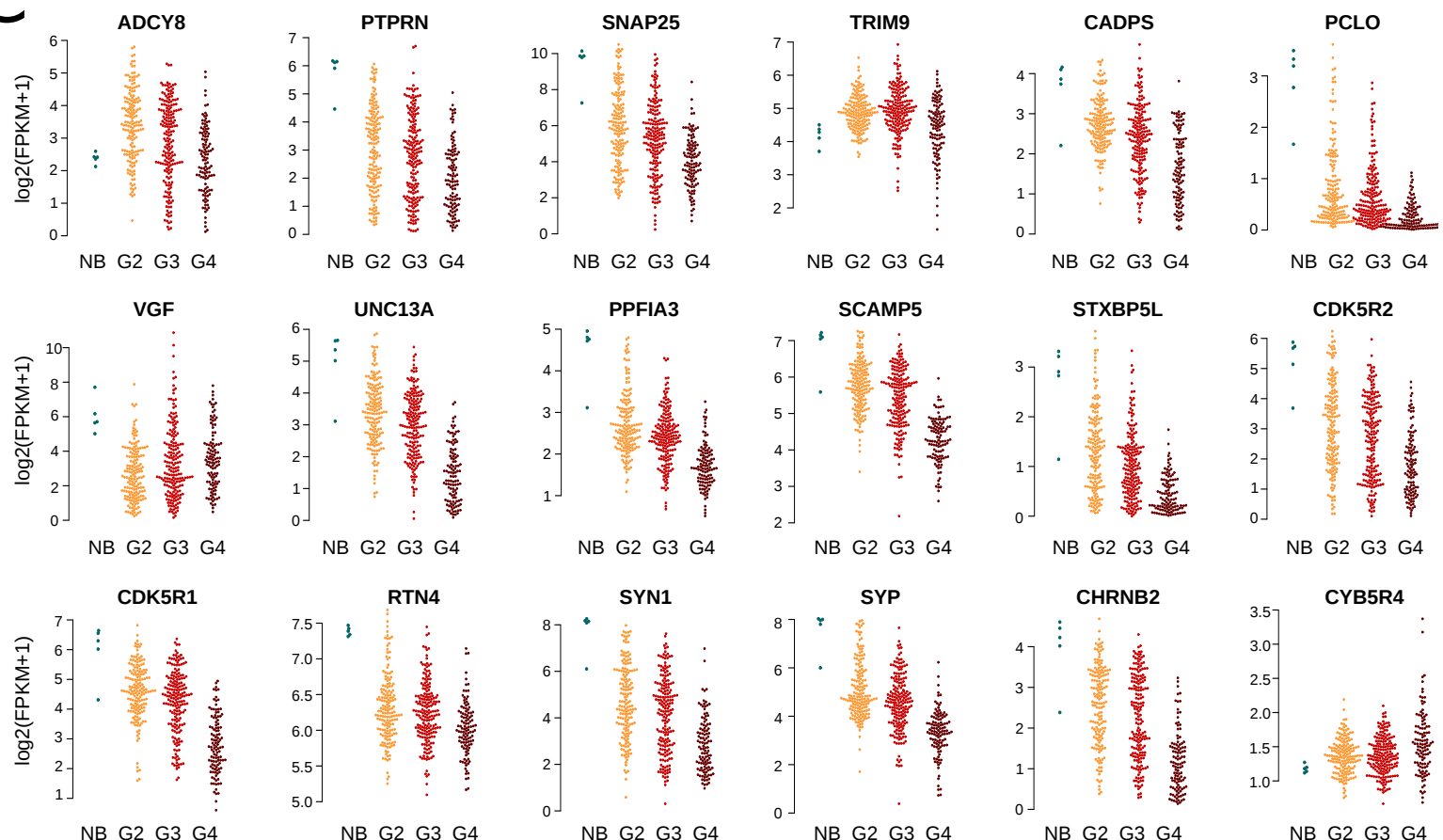

Supplement: Supplementary file 7 — Additional file 7. GO Biological Pathway enrichment for the genes identified in REST ChIP-seq and and REST knockdown experiments. A GO BP analysis for the genes that were upregulated or B downregulated by siREST and targeted by REST in the ChIP-seq experiment. ChIP-seq experiments on both U87 IDH-MUT, IDH-WT cell lines and from freshly resected human tumors show a good overlap of the number of genes annotated to ChIP-seq peaks. C Expression of genes upregulated in REST depleted cells (from panel A) in TCGA LGG/GBM datasets and presented as bee swarm plots for NB (normal brain), glioma WHO G2, G3 and G4. [file 40478_2024_1779_MOESM7_ESM.pdf]

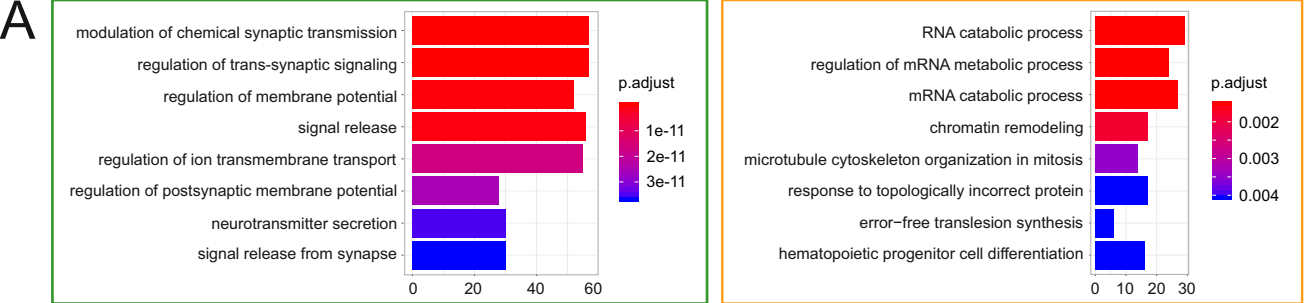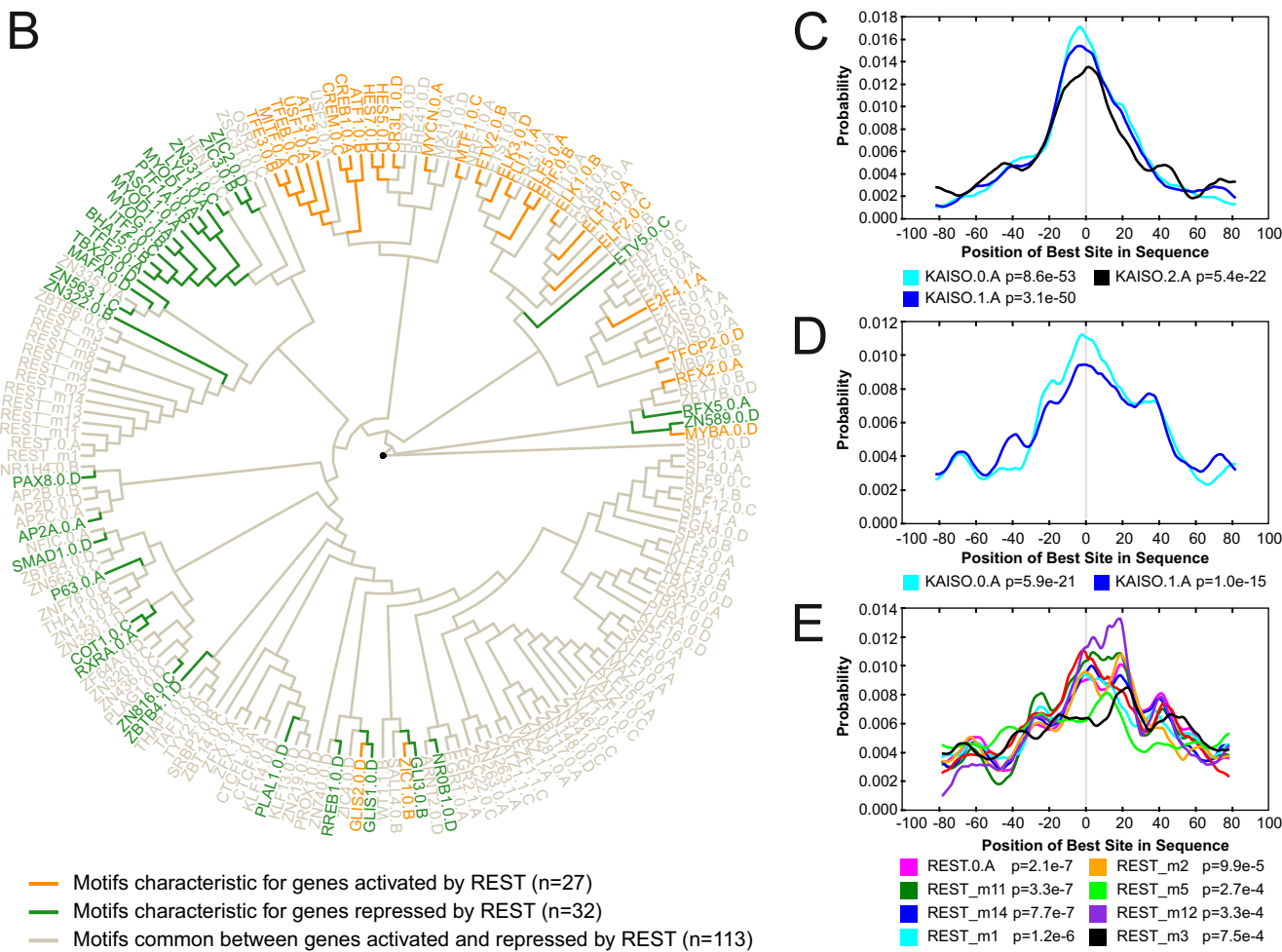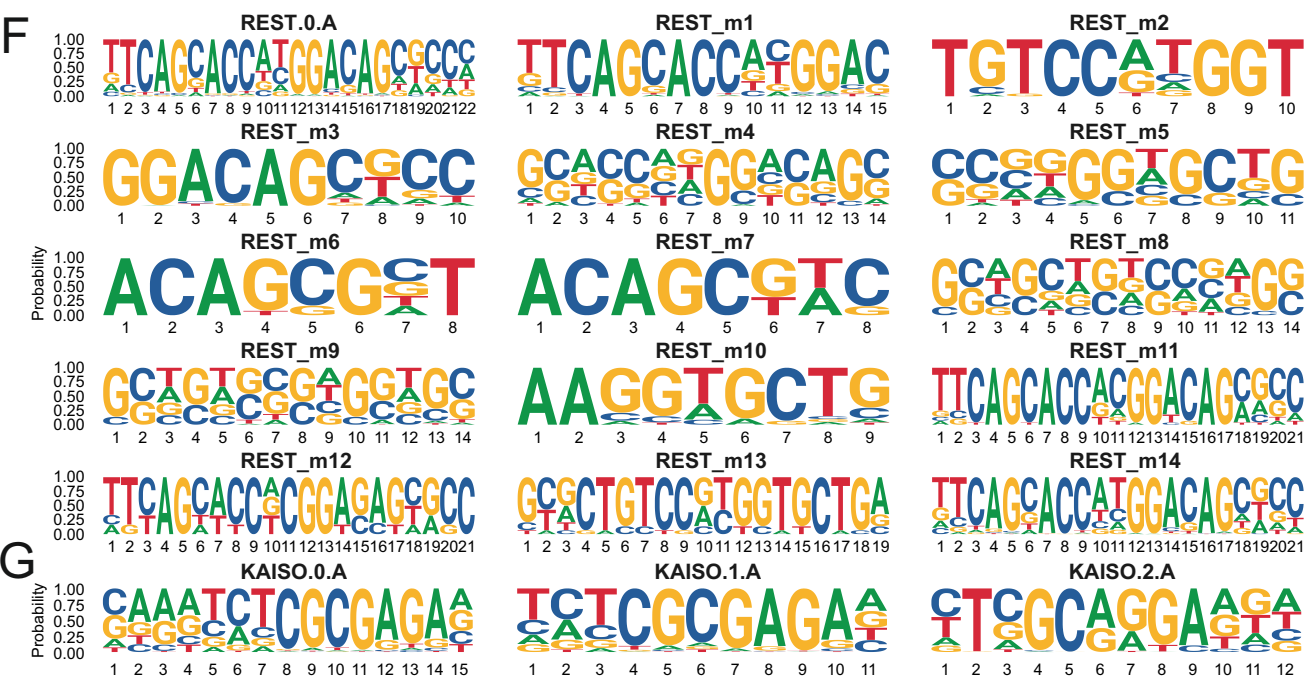

Supplement: Supplementary file 8 — Additional file 8. Characterization of ChIP-seq peaks and TF motifs in REST-repressed and REST-activated genes. A Gene Ontology Biological Process gene enrichment analysis for repressed REST targets (left panel) and activated REST targets (right panel). B Clustering of TF motifs characteristic for genes activated by REST, repressed by REST, and common to both REST-activated and REST-repressed, based on TF PWMs. Distribution of KAISO motifs within the promoters of the genes activated (C) and repressed (D) by REST. E Distribution of REST motifs within the promoters of the genes repressed by REST. F DNA sequence logos of the REST binding motifs PWMs; G DNA sequence logos of the KAISO binding motifs PWMs. [file 40478_2024_1779_MOESM8_ESM.pdf]

A

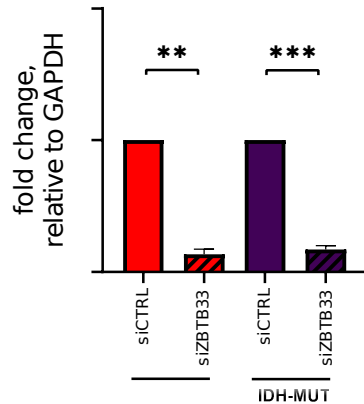

B

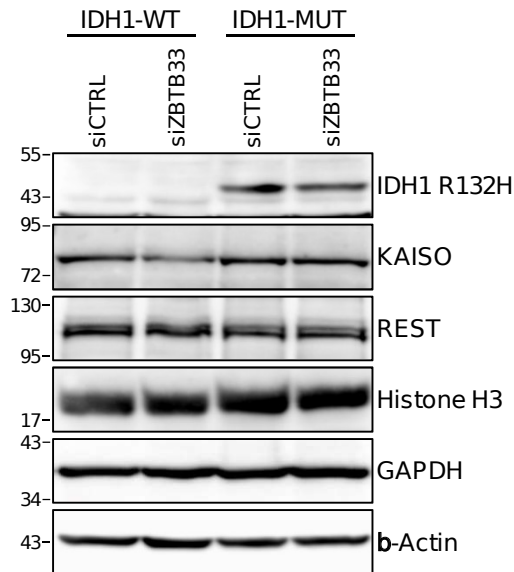

C

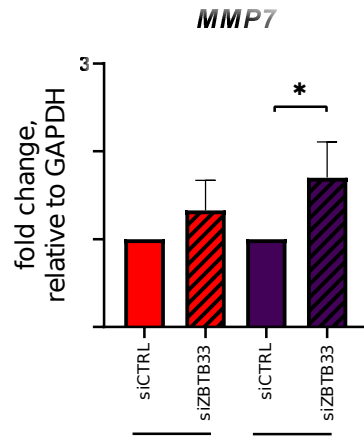

Supplement: Supplementary file 9 — Additional file 9. ZBTB33 (KAISO) silencing experiment. A Relative expression of ZBTB33 (coding KAISO) in IDH-WT and IDH-MUT U87 cells at 72 hours of silencing with siRNA. mRNA levels in transfected cells were determined by quantitative PCR and normalized to GAPDH expression in the same sample, and expressed as the fold change relative to the siCTRL. Data are represented as mean ± SD, n = 4 independent experiments, **p<0.01, ***p<0.001, one way ANOVA. B Representative immunoblots illustrating levels of KAISO and reference proteins (mutated IDH1, REST, Histone H3, GAPDH and beta-actin) in IDH-WT and IDH-MUT U87 cells at 72 hours after knockdown of KAISO determined by Western blotting. C Relative expression of MMP7 in IDH-WT and IDH-MUT U87 cells at 72 hours of ZBTB33 (coding KAISO) silencing with siRNA. mRNA levels in transfected cells were determined by quantitative PCR and normalized to GAPDH expression in the same sample and expressed as the fold change relative to the siCTRL. Data are represented as mean ± SD, n = 4 independent experiments, *p<0.05, one way ANOVA. [file 40478_2024_1779_MOESM9_ESM.pdf]
