# Supplementary material for: Comprehensive analysis of the REST transcription factor regulatory networks in IDH mutant and IDH wild-type glioma cell lines and tumors
Source: Acta Neuropathol Commun. 2024 May 6;12:72. doi: 10.1186/s40478-024-01779-y (PMC11071216; doi:10.1186/s40478-024-01779-y)
Supplement: Supplementary file 4 — Additional file 4. REST ChIP-seq peaks DNA methylation level, of U87 IDH-WT and U87 IDH-MUT cell lines samples treated with siCTRL and siREST, in a resolution of single cytosine loci. List of REST repressed or REST activated genes containing differentially methylated sites in REST or KAISO motifs within the associated REST ChiPseq peaks. A Difference versus log10 fold change (FC) in DNA methylation between siREST and siCTRL samples of U87 IDH-WT cell line; B as in A but for U87 IDH-MUT cell line; C Difference versus FC in DNA methylation between siCTRL IDH-MUT and siCTRL WT samples; D as in C but for siREST IDH-MUT and siREST IDH-WT samples; In A-D: light gray - no difference, red - decreased methylation (difference ≤ − 0.15), green - increased methylation (difference ≥ 0.15); E Difference in cytosine methylation between U87 IDH-MUT and U87 IDH-WT cell lines in siREST and siCTRL samples; F Cytosines within REST ChIP-seq peaks were divided into three categories (WT-specific, MUT-specific, common) and presented as percentage of single cytosines that overlapped with one of the three types of REST ChIP-seq peaks. “Background” refers to all cytosines within REST-peaks, “Differential” represents non-light gray cytosines shown C&D, “Higher” (red) - cytosines with higher DNA methylation in IDH-MUT, "Lower" (green) - cytosines with lower DNA methylation in IDH-MUT; G log10 fold change between percentage of “Differential” cytosines to “Background” cytosines in each of the three REST ChIP-seq peaks categories: common, WT-, MUT-specific. [file 40478_2024_1779_MOESM4_ESM.pdf]

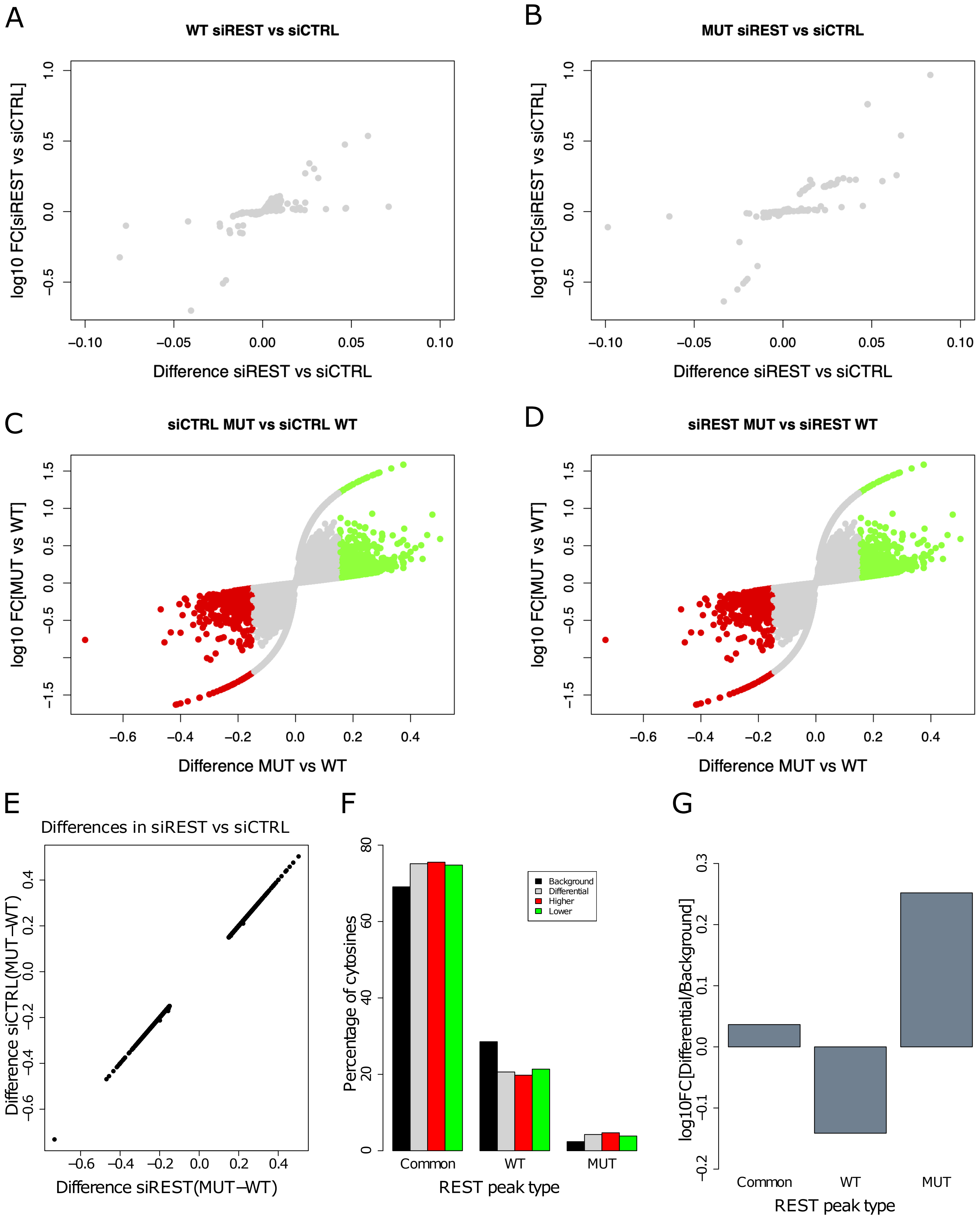

A
WT siREST vs siCTRL
log10 FC[siREST vs siCTRL]
Difference siREST vs siCTRL
B
MUT siREST vs siCTRL
log10 FC[siREST vs siCTRL]
Difference siREST vs siCTRL
C
siCTRL MUT vs siCTRL WT
log10 FC[MUT vs WT]
Difference MUT vs WT
D
siREST MUT vs siREST WT
log10 FC[MUT vs WT]
Difference MUT vs WT
E
Differences in siREST vs siCTRL
Difference siCTRL(MUT−WT)
Difference siREST(MUT−WT)
F
Percentage of cytosines
Background
Differential
Higher
Lower
Common
WT
MUT
REST peak type
G
log10FC[Differential/Background]
Common
WT
MUT
REST peak type
