# Supplementary material for: Comprehensive analysis of the REST transcription factor regulatory networks in IDH mutant and IDH wild-type glioma cell lines and tumors
Source: Acta Neuropathol Commun. 2024 May 6;12:72. doi: 10.1186/s40478-024-01779-y (PMC11071216; doi:10.1186/s40478-024-01779-y)
Supplement: Supplementary file 10 — Additional file 10. REST and KAISO silencing additional information. A Bootstrapping results for siKAISO experiment, where number of siKAISO DEGs was intersected with REST-repressed and REST-activated gene categories separately in IDH-MUT and IDH-WT samples (red dots) and compared to random sampling experiment were the same number of genes as number of genes in REST-activated or REST-repressed was drawn 1000 times and checked how many of them appeared as DEGs in our siKAISO experiment (gray dots). B The same bootstrapping experiment as described above but for siREST experiment. C Venn diagrams of genes up- or downregulated by siREST treatment in IDH-WT or IDH-MUT cells. D Venn diagrams of genes up- or downregulated by siKAISO treatment in IDH-WT or IDH-MUT cells. E Plots of 6 last deciles (out of 10 total) of log2 of variance from normalized counts for siCTRL for IDH-MUT and IDH-WT and siREST IDH-MUT and IDH-WT. F Plots of 6 last deciles (out of 10 total) of log2 of variance from normalized counts for siCTRL for IDH-MUT and IDH-WT and siKAISO IDH-MUT and IDH-WT. [file 40478_2024_1779_MOESM10_ESM.pdf]

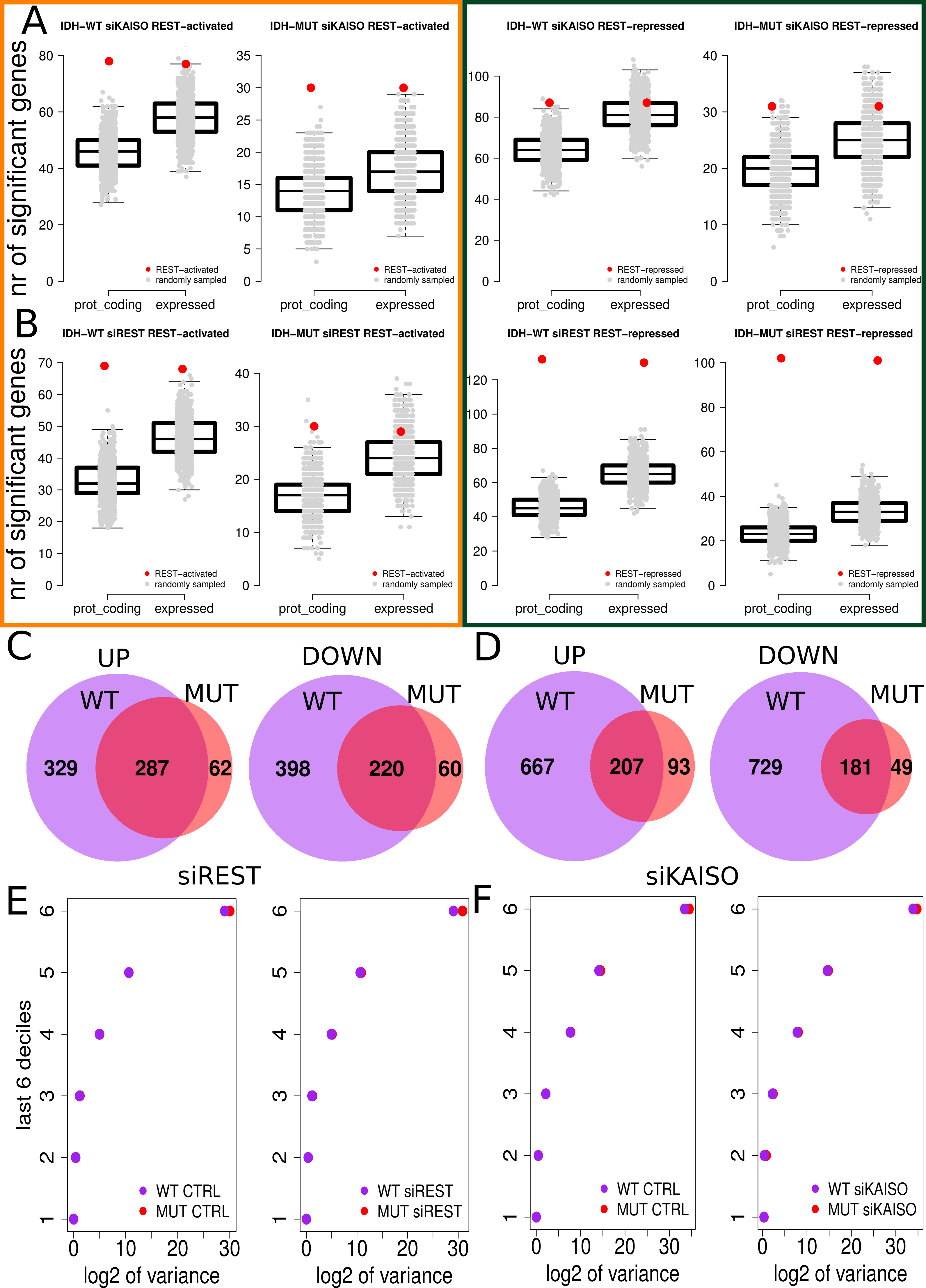

A
B
C
D
E
F
IDH−WT siKAISO REST−activated
IDH−MUT siKAISO REST−activated
IDH−WT siKAISO REST−repressed
IDH−MUT siKAISO REST−repressed
IDH−WT siREST REST−activated
IDH−MUT siREST REST−activated
IDH−WT siREST REST−repressed
IDH−MUT siREST REST−repressed
nr of significant genes
prot_coding
expressed
REST−activated
REST−repressed
randomly sampled
UP
DOWN
WT
MUT
329
287
62
398
220
60
667
207
93
729
181
49
siREST
siKAISO
last 6 deciles
log2 of variance
WT CTRL
MUT CTRL
WT siREST
MUT siREST
WT siKAISO
MUT siKAISO
